# Shards, sequences, and shorelines: two new species of *Bembidion* from North America (Coleoptera, Carabidae)

**DOI:** 10.3897/zookeys.1007.60012

**Published:** 2020-12-30

**Authors:** David R. Maddison

**Affiliations:** 1 Department of Integrative Biology, Oregon State University, Corvallis, OR 97331, USA Oregon State University Corvallis United States of America

**Keywords:** Bembidiini, ground beetle, molecular systematics, species delimitation, taxonomy, Trechinae

## Abstract

Two new species of *Bembidion* are described from river shores in North America. One, *Bembidion mimbres***sp. nov.**, from the Gila River watershed in the lands of the Mimbres culture in New Mexico and Arizona, is closely related to the widespread *Bembidion levigatum*. DNA sequences from several linkage groups and morphology provide evidence of the distinctiveness of *B. mimbres*. The second, *Bembidion corgenoma***sp. nov**., has been the subject of recent genomic and transcriptomic studies. It belongs in the *Bembidion transversale* subgroup, and occurs from California north to British Columbia, east to Montana and Nevada. The *B. transversale* subgroup as a whole is reviewed, and morphological characters that distinguish *B. corgenoma* from the similar and sympatric *B. transversale* and *B. erosum* are described and illustrated. DNA sequences of these three species show no consistent differences in 28S, COI, CAD, and Topoisomerase, and a coalescent species delimitation analysis reveals no notable structure within the complex. This is the first known trio of species within *Bembidion* for which those genes provide no clear signal of species boundaries. A neotype is designated for the one name in the group that lacks a primary type, *Bembidium haplogonum* Chaudoir. Chromosomes of the new species and their relatives are as is typical for *Bembidion*, with eleven pairs of autosomes and an XY/XX sex chromosome system.

## Introduction

*Bembidion* is a very large genus of small beetles with more than 1,200 species worldwide (Lorenz 2005). Most species of these small predators live along the edges of bodies of water and can be abundant in their habitats.

In the course of an ongoing project revising the bembidiine carabids of America north of Mexico, a number of undescribed species have been discovered. Most of these will be described in due course within complete revisions of subgenera or species groups. However, two of these new species are or will be soon discussed in the scientific literature, and warrant description more quickly, in order to provide them with names. These two are also especially significant, as they have cultural connections to humans, implicit or explicit, of very different sorts.

**Figure 1.**
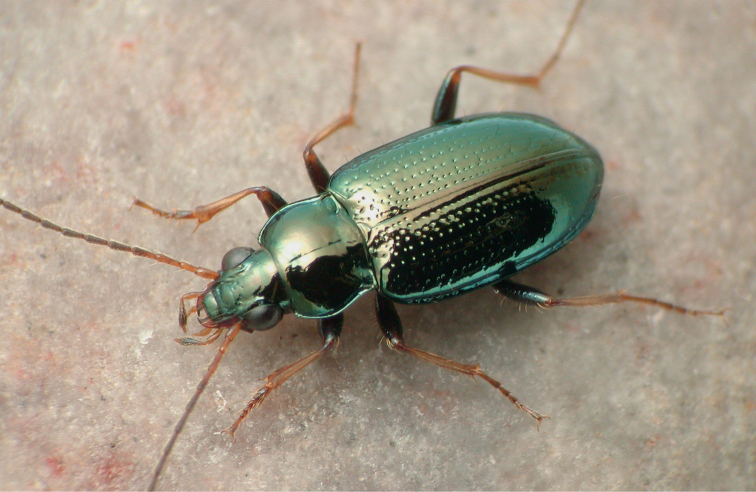
Paratype male of *Bembidion mimbres* (voucher number V100327) from the type locality.

**Figure 2.**
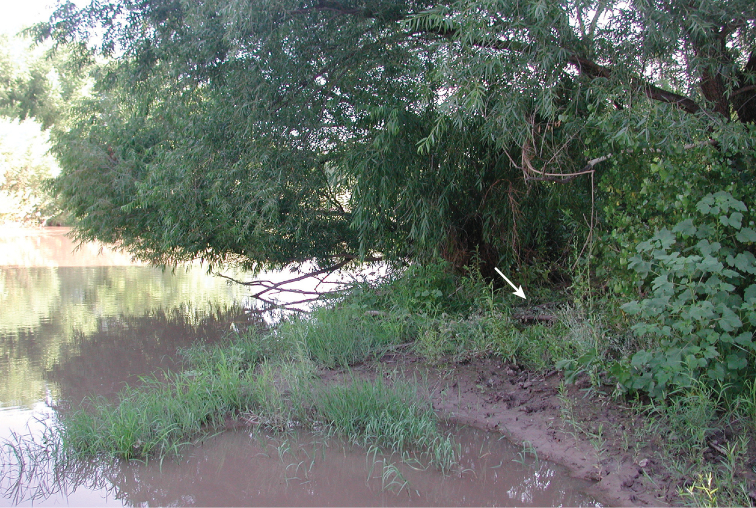
Habitat of *Bembidion mimbres* at USA: New Mexico: Grant Co., Gila River, Billings Vista, 1320 m, 32.8163°N, 108.6032°W (type locality). Arrow indicates approximate location of most specimens. Found in the same habitat were *Bembidion aratum* (LeConte), *B. impotens* Casey, *B. scintillans* Bates, *B. horni* Hayward, *B. rupicola* (Kirby), *B. clemens* Casey, *B rapidum* (LeConte), and *Omophron obliteratum* Horn.

One of them is a member of the subgenus Hydrium, a group of relatively large *Bembidion* that is widespread in the Northern Hemisphere. The new species (Fig. 1) is only known from the Gila River watershed of southeastern Arizona and southwestern New Mexico, where it lives along the banks of rivers and creeks, on the ground a few meters away from the shoreline (Fig. 2), most commonly under willows (*Salix*). The distribution of this new species is within that of the Mimbres culture, which flourished in that area one thousand years ago. This culture is perhaps best known for black-on-white Mimbres pottery, the designs of which depicted people, cultural icons, and organisms (Hegmon et al. 2018). The people of the Mimbres culture were deeply aware of the arthropods in their environment, as indicated by the astonishing array of images on their pottery of insects, including among others geometrid larvae (https://core.tdar.org/image/383483/1452-style-iii-bowl-from-cameron-creek; https://doi.org/10.6067/XCV8Z60P2N), Orthoptera (https://core.tdar.org/image/383111/2685-style-iii-bowl-from-swarts; https://doi.org/10.6067/XCV80Z7364), dragonflies, and ant lions (Hegmon, et al. 2018). In honor of these peoples who were so connected to the small organisms in their midst, this elegant beetle species is given the name *Bembidion mimbres*.

**Figure 3.**
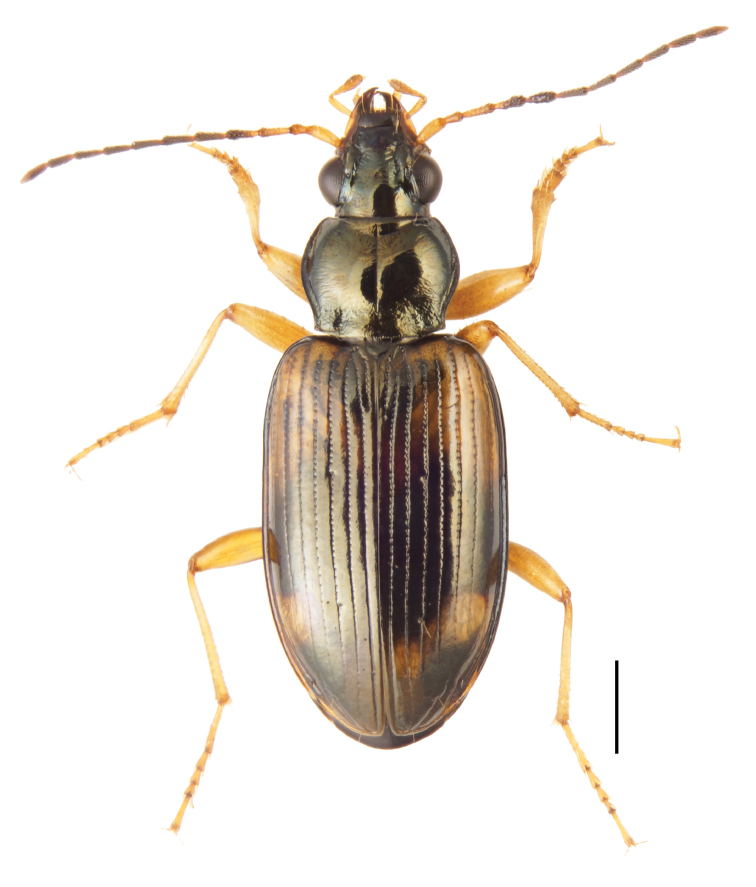
Paratype male of *Bembidion corgenoma* (voucher number V101452) from the type locality. Scale bar: 1 mm.

**Figure 4.**
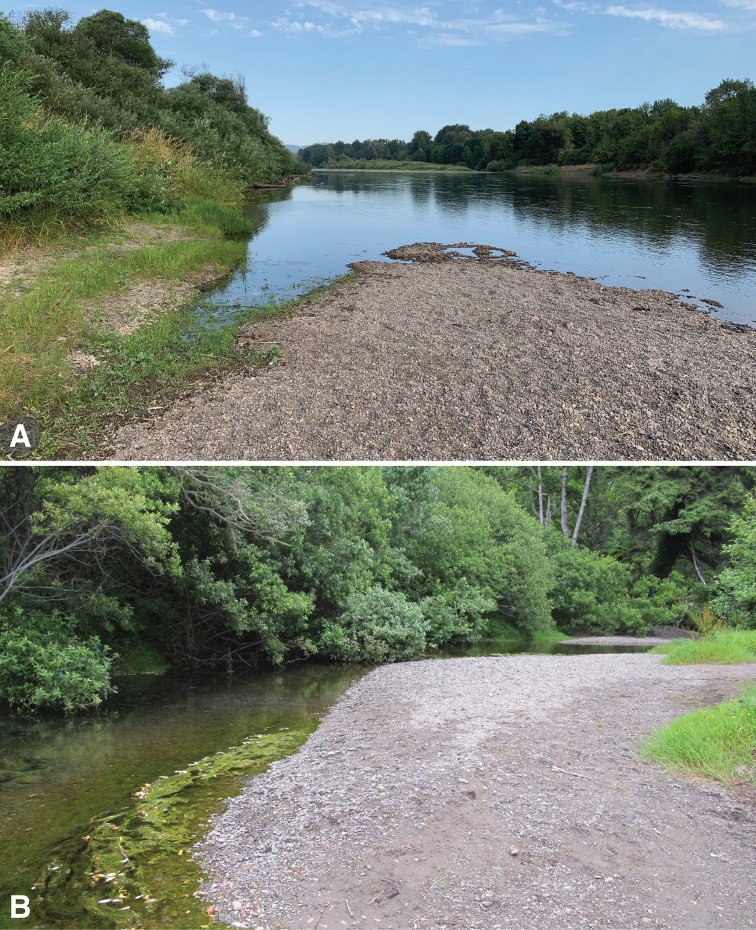
Habitats of *Bembidion corgenoma***A** USA: Oregon: Benton Co., Corvallis, Willamette River, 60 m, 44.5491°N, 123.2449°W (type locality). *B. corgenoma* and *B. recticolle* are both common in this habitat. *B. corgenoma* is more common near the water in areas with vegetation growing nearby among the gravel and cobbles **B** USA: California: Del Norte Co., Wilson Creek, 3 m, 41.6051°N, 124.1005°W, habitat of *B. corgenoma* and *B. erosum*, as well as *Bembidion haruspex* Casey, *B. vandykei* Blaisdell, *B. curtulatum* Casey, *B. platynoides* Hayward, and *B incrematum* LeConte.

The second species described here is connected to humans via modern biological research: it is becoming the first model species of *Bembidion* for genomic and transcriptomic studies. It is member of the *B. transversale* species group of the *Ocydromus* complex of *Bembidion*, containing some of the largest *Bembidion* in North America (Lindroth 1963; Maddison and Swanson 2010). The *B. transversale* group consists of two subgroups, the *B. transversale* subgroup and the *B. mexicanum* subgroup (Maddison 2012; Maddison and Swanson 2010). Maddison and Swanson (2010) considered the *B. transversale* subgroup to contain three species (*B. transversale* Dejean, *B. perspicuum* (LeConte), and *B. sarpedon* Casey), although they noted that “*B. transversale*” showed enough morphological variation to suggest that it may contain multiple species. Because of the especially complex pattern of variation, it only recently become clear that the genomic and transcriptomic model species was undescribed. The new species (Fig. 3) is common in Oregon and California, with some populations in neighboring regions, living along cobble and gravel shores of rivers and creeks (Fig. 4). It is the best sequenced *Bembidion* genomically and transcriptomically (Gustafson et al. 2019; Gustafson et al. 2020; Pflug et al. 2020), and has been used as one of the models for developing a UCE probe set for adephagan beetles (Gustafson, et al. 2019; as *B. haplogonum* Chaudoir). It is a centerpiece of ongoing and future studies of genome size in carabids (e.g., Pflug, et al. 2020). In this paper, it is described as *Bembidion corgenoma*.

An important step enabling future research about these beetles is providing the species with stable names. Although we now know the two new species in many ways unimaginable to those who lived a thousand years ago, including detailed aspects of their DNA and genomes, we know very little about these two species in nature. With the decreasing emphasis on natural history in modern biology, it is possible that a person of the Mimbres culture knew aspects of the daily life of *B. mimbres* much better than we ever will. However, perhaps this paper, in giving names to the two species and presenting identification tools allowing them and their near relatives to be distinguished, will inspire research about these beetles, including into their way of life along river shores.

## Materials and methods

Members of *Bembidion* were examined from or will be deposited in the collections listed below. Each collection’s listing begins with the code used in the text.

**CAS**California Academy of Sciences, San Francisco, USA;

**CNC**Canadian National Collection, Ottawa, Canada;

**CSCA**California State Collection of Arthropods, Sacramento, USA;

**EMEC**Essig Museum Entomology Collection, University of California, Berkeley, USA;

**MCZ**Museum of Comparative Zoology, Harvard University, Cambridge, USA;

**NHMUK**The Natural History Museum, London, UK;

**MNHN**Muséum National d’Histoire Naturelle, Paris, France;

**MSBA**Museum of Southwestern Biology, University of New Mexico, Albuquerque, USA;

**OSAC**Oregon State Arthropod Collection, Oregon State University, Corvallis, USA;

**UAIC**University of Arizona Insect Collection, Tucson, USA;

**UASM**University of Alberta Strickland Museum, Edmonton, Canada;

**UBCZ**Spencer Entomological Museum, University of British Columbia, Vancouver, Canada;

**USNM**National Museum of Natural History, Smithsonian Institution, Washington, DC, USA;

**ZMUM**Zoological Museum, Moscow State University, Moscow, Russia.

### Collecting methods

Specimens were collected by hand or using an aspirator; specimens were found during the day in their habitat, or with the aid of a headlamp at night, when the beetles are more actively moving on the surface. Specimens for morphological studies were killed and preserved in maple (*Acer*) sawdust to which ethyl acetate was added. Specimens for DNA sequencing were collected into 95% or 100% ethanol. For chromosomal studies, live specimens were placed in simple Carnoy’s solution (three parts 100% ethanol : one part glacial acetic acid), and the abdomens were opened up shortly after death to allow better penetration of the fixative; the specimens were stored in Carnoy’s in a -20 °C freezer, with vials contained within multiple layers of plastic to prevent the release of acid fumes.

### Morphological methods

General methods of specimen preparation for morphological work, and terms used, follow Maddison (1993; 2008). Genitalia were prepared, after dissection from the body, by treatment in 10% KOH at 65 °C for 10 minutes followed by a series of multi-hour baths of distilled water, 5% glacial acetic acid, distilled water, and then ethanol. Male genitalia were then mounted in Euparal between two small coverslips attached to archival-quality heavyweight watercolor paper, and, once dried, pinned beneath the specimen. Male genitalia were examined for 30–60 specimens each of *B. transversale*, *B. erosum*, and *B. corgenoma*, and four or five specimens each of *B. levigatum* and *B. mimbres*.

Photographs of entire beetles and antennae were taken with a Leica M165C dissecting scope and a Sony NEX-7 camera, and of male genitalia with either a Leica Z6 Apo lens and DMC4500 camera or a Leica DM5500B compound microscope and DMC425C camera. A stack of images from different focal positions was then merged using the PMax procedure in Zerene Systems’s Zerene Stacker; the final images thus potentially have some artifacts caused by the merging algorithm. Measurements were made using Leica Application Suite v4.9 from images acquired using these imaging systems.

### Cytogenetic methods

Twenty-two males were examined for chromosome number and sex-chromosome system. Methods used were as outlined by Maddison (1985; 2008). Males examined were: 1 *Bembidion mimbres* from USA: New Mexico: Grant Co., Billings Vista, Gila River, 1310 m, 32.8137°N, 108.6031°W; 2 *B. transversale* from USA: Colorado: Fremont Co., Arkansas River at Texas Creek 1880 m, 38.4100°N, 105.5854°W; 3 *B. erosum* from USA: California: Del Norte Co., Wilson Creek , 3 m, 41.6051°N, 124.1005°W; 3 *B. perspicuum* from USA: Arizona: Cochise Co., San Pedro R at Charleston, 31.6239°N, 110.1722°W; 3 *B. sarpedon* from USA: Colorado: Las Animas Co., Purgatoire R., 2.7 km W Cokedale 1910 m, 37.1295°N, 104.6390°W; 4 *B. pernotum* from USA: Colorado: Las Animas Co., Purgatoire R., 2.7 km W Cokedale 1910 m, 37.1292°N, 104.6398°W; 3 *B. mexicanum* from USA: Arizona: Pima Co., Santa Rita Mtns, Box Canyon, 1455 m 31.7981°N, 110.7767°W; 3 *B. lugubre* from USA: Arizona: Pima Co., Santa Rita Mtns, Box Canyon, 1455 m, 31.7981°N, 110.7767°W. In addition, the vouchers of the *B. transversale* group studied in Maddison (1985) were re-examined and re-identified.

### Molecular methods

#### DNA extraction and sequencing

Genes studied, and abbreviations used in this paper, are: 28S: 28S ribosomal DNA (D1-D3 domains); 18S: 18S ribosomal DNA; COI: cytochrome *c* oxidase I; *wg*: *wingless*; CAD: carbamoyl phosphate synthetase domain of the *rudimentary* gene; ArgK: arginine kinase; Topo: topoisomerase I.

DNA was extracted using a Qiagen DNeasy Blood and Tissue Kit. Fragments for the seven genes were amplified using the polymerase chain reaction (PCR) on an Eppendorf Mastercycler ProS Thermal Cycler, using TaKaRa Ex Taq and the basic protocols recommended by the manufacturers. Primers and details of the cycling reactions used are given in Maddison (2012) and Maddison and Cooper (2014). The amplified products were then cleaned, quantified, and sequenced at the University of Arizona’s Genomic and Technology Core Facility using a 3730 XL Applied Biosystems automatic sequencer. Assembly of multiple chromatograms for each gene fragment and initial base calls were made with Phred (Green and Ewing 2002) and Phrap (Green 1999) as orchestrated by Mesquite’s Chromaseq package (Maddison and Maddison 2018a, c) with subsequent modifications by Chromaseq and manual inspection. Multiple peaks at a single position in multiple reads were coded using IUPAC ambiguity codes.

#### Taxon sampling for DNA studies

For the phylogenetic study of Bembidion (Hydrium), 19 specimens of the subgenus Hydrium, as well as five species serving as outgroups (Table 1) were used. Of the 152 sequences examined, 97 were newly acquired, with 55 being from previous publications (Maddison 2012; Maddison and Cooper 2014; Maddison et al. 2019; Maddison and Maruyama 2019; Maddison and Ober 2011). For the phylogenetic study of the *B. transversale* group, I sampled 56 specimens of the *B. transversale* subgroup, as well as three species of the *B. mexicanum* subgroup (Table 2). Of the 237 sequences examined, 109 were newly sequenced, with 128 from previous publications (Kanda et al. 2015; Maddison 2012; Maddison and Swanson 2010; Wild and Maddison 2008). Sequences of the two holotypes listed in Tables 1 and 2 are “genseq-1”, of paratypes “genseq-2”, and the remainder are all “genseq-4” (Chakrabarty et al. 2013). In addition, sequences of the newly designated neotype of *Bembidium haplogonum* Chaudoir were acquired and deposited in GenBank (accession numbers MW151478, MW151506, and MW151563), and are “genseq-1”. Localities of the sequenced specimens of *B. levigatum*, *B. mimbres*, *B. transversale*, *B. erosum*, and *B. corgenoma* are given in Table 3.

**Table 1.** Sampling of members of Bembidion (Hydrium) and related subgenera for DNA-based study. Four-digit numbers under “#” are D.R. Maddison DNA voucher numbers; the specimen marked with an * is the holotype of *B. mimbres*. All other specimens listed of *B. mimbres* are paratypes. An abbreviation for state or province of capture is given under “Loc”; further information on *B. levigatum* and *B. mimbres* specimens is given in Table 3. The last eight columns contain GenBank accession numbers.

	#	Loc	28S	COIBC	COIPJ	COI-like	wg	CAD	ArgK	Topo
*** Outgroups ***										
*B. incisum* Andrewes			JN170312	JN171013			JN171381	MK118269	JN170532	JN171195
*B. dyschirinum* LeConte	0896		JN170332	JN171029	MW151370		GU556029	JN170799	JN170553	JN171210
*B. lampros* (Herbst)	1727		JN170370	MF616889	MW151371		JN171439	JN170838	JN170593	MF616755
*B. properans* (Stephens)	1279		JN170408	JN171088	MW151372		JN171482	JN170883	JN170640	JN171270
*B. luridicorne* Solsky	1122		JN170377	JN171064	MW151373		JN171447	JN170847	JN170602	JN171245
**Bembidion (Hydrium)**										
*B. interventor* Lindroth	1131		JN170365	JN171052	MW151374		JN171434	JN170833	JN170588	JN171232
*B. nitidum* (Kirby)	1941		JN170392	MF616897	MW151375		JN171464	JN170864	JN170620	JN171257
*B. obliquulum* LeConte	1299		JN170395	KJ624355	MW151376		JN171467	MK118221	JN170623	KJ624308
*B. levigatum* Say	1693	VA	MW151391	JN171059	JN171059		JN171441	JN170841	JN170596	JN171240
	1255	IN	MW151392	MW151377	MW151377			MW151435		MW151406
	1694	IA	MW151393	MW151378	MW151378		MW151428	MW151436	MW151420	MW151407
	0763	NE	GU556083	MW151379	MW151379		GU556032			
	1256	TX	MW151394	MW151380	MW151380			MW151437	MW151421	MW151408
	2343	TX	MW151395	MW151381	MW151381		MW151429	MW151438	MW151422	MW151409
*B. mimbres* sp. nov.	0280	NM	MW151396	MW151382	MW151382		MW151430	MW151439	MW151423	MW151410
	1220	NM	MW151404			MW151366		MW151447		MW151418
	1267	NM	MW151405			MW151367		MW151448		MW151419
	1944	NM	MW151403	MW151389		MW151368	MW151434	MW151446	MW151427	MW151417
	2117	NM	MW151397	MW151383	MW151383	MW151369		MW151440		MW151411
	2118	NM	MW151398	MW151384	MW151384		MW151431	MW151441	MW151424	MW151412
	2119	NM	MW151399	MW151385	MW151385			MW151442		MW151413
	2131*	NM	MW151400	MW151386	MW151386		MW151432	MW151443	MW151425	MW151414
	2134	NM	MW151401	MW151387	MW151387			MW151444		MW151415
	2135	NM	MW151402	MW151388	MW151388		MW151433	MW151445	MW151426	MW151416

**Table 2.** Sampling of members of *Bembidion transversale* group for DNA-based study. Four-digit numbers under “#” are D.R. Maddison DNA voucher numbers. Under “T”, the holotype of *B. corgenoma* is indicated by “H”, and paratypes by “P”. An abbreviation for state or province of capture is given under “Loc”; further information on specimens of *B. transversale*, *B. erosum*, and *B. corgenoma* is given in Table 3. The last four columns contain GenBank accession numbers.

	#	T	Loc	28S	COI	CAD	Topo
***B. mexicanum* subgroup**							
*Bembidion lugubre* LeConte	1712		AZ	JN170375	JN171062	JN170845	JN171243
*Bembidion mexicanum* Dejean	2192		NM	GU454739	GU454769	JN170854	JN171250
*Bembidion pernotum* Casey	2483		CO	JN170403	JN171082	JN170875	JN171263
***B. transversale* subgroup**							
*Bembidion sarpedon* Casey	2484		CO	JN170432	JN171106	JN170908	JN171285
	2514		CO	KU233761	KU233815	KU233926	KU234052
	3009		CO	KU233764	KU233818	KU233929	KU234055
	3761		UT	KU233771	KU233823	KU233933	KU234059
	3776		UT	KU233775	KU233827	KU233937	KU234063
*Bembidion perspicuum* (LeConte)	1120		AZ	GU454740	GU454770	JN170877	JN171265
	2318		CA	GU454743	KU233812	KU233923	KU234049
	3774		CA	KU233773	KU233825	KU233935	KU234061
	3775		CA	KU233774	KU233826	KU233936	KU234062
	2485		CO	GU454748	GU454778	KU233924	KU234050
	2182		NM	GU454749	KU233810	KU233922	KU234047
*Bembidion erosum* Motschulsky	2596		CA	MW151550	MW151493	MW151522	MW151465
	2607		CA	MW151551	MW151494	MW151523	MW151466
	3561		CA	MW151552	MW151495	MW151524	MW151467
	3562		CA	MW151553	MW151496	MW151525	MW151468
	3584		CA	MW151554	MW151497	MW151526	MW151469
	4050		CA	MW151549	MW151492	MW151521	MW151464
	4212		CA	MW151555	MW151498	MW151527	MW151470
	4033		OR	MW151556	MW151499	MW151528	MW151471
	2162		BC	KU233749	KU233803	KU233915	KU234040
	2194		BC	KU233757	KU233811	MW151529	KU234040
*Bembidion transversale* Dejean	2160		NS	GU454762	KU233802	KU233914	KU234048
	2486		CO	GU454755	GU454785	KU233925	KU234039
	2157		WY	EU677688	GU454797	EU677541	KU234051
	4690		MT	MW151561	MW151504	MW151534	MW151476
	4927		MT	MW151562	MW151505	MW151535	MW151477
	5064		WA	MW151560	MW151503	MW151533	MW151475
	5613		OR	MW151557	MW151500	MW151530	MW151472
	4219		OR	MW151558	MW151501	MW151531	MW151473
	5612		OR	MW151559	MW151502	MW151532	MW151474
*Bembidion corgenoma* sp. nov.	4052		CA	KU233782	KU233831	KU233941	KU234067
	2181		CA	KU233755	KU233809	KU233921	KU234046
	4054		CA	KU233783	KU233832	KU233942	KU234068
	2180		CA	KU233754	KU233808	KU233920	KU234045
	3772	P	CA	KU233772	KU233824	KU233934	KU234060
	4961	P	CA	MW151536	MW151479	MW151508	MW151451
	4962	P	CA	MW151537	MW151480	MW151509	MW151452
	4218		CA	MW151544	MW151487	MW151516	MW151459
	2597		CA	MW151538	MW151481	MW151510	MW151453
	2608		CA	MW151539	MW151482	MW151511	MW151454
	3559		CA	KU233769	KU233821	KU233931	KU234057
	3560		CA	KU233770	KU233822	KU233932	KU234058
	3583		CA	MW151540	MW151483	MW151512	MW151455
	4959		NV	MW151541	MW151484	MW151513	MW151456
	2346		NV	GU454763	GU454793	MW151507	MW151450
	5670	P	OR	MW151545	MW151488	MW151517	MW151460
*Bembidion corgenoma* sp. nov.	5671	P	OR	MW151546	MW151489	MW151518	MW151461
	5672	P	OR	MW151547	MW151490	MW151519	MW151462
	5673	H	OR	MW151548	MW151491	MW151520	MW151463
	4032		OR	KU233780	KU233829	KU233939	KU234065
	2973		OR	KU233763	KU233817	KU233928	KU234054
	3205	P	OR	KU233791	KU233841	KU233979	KU234056
	4034		OR	KU233781	KU233830	KU233940	KU234066
	3021		ID	KU233790	KU233840	KU233973	KU234070
	2165		WA	KU233750	KU233804	KU233916	KU234041
	5065	P	OR	MW151542	MW151485	MW151514	MW151457
	2190		BC	MW151543	MW151486	MW151515	MW151458

**Table 3.** Localities of capture of *Bembidion* specimens of *B. levigatum*, *B. mimbres*, and the *B. transversale* subgroup whose DNA was sequenced. Four-digit numbers at the start of each row are D.R. Maddison DNA voucher numbers.

***Bembidion levigatum* Say**	
**0763**	USA: Nebraska: Lancaster Co., Lincoln, Wilderness Park along Salt Creek, 360 m 40.6983°N, 96.6837°W
**1255**	USA: Indiana: Crawford Co., Ohio River near Schooner‘s Point, 120 m 38.1571°N, 86.3379°W
**1256**	USA: Texas: Somervell Co., Brazos River and Route 67, 175 m 32.2694°N, 97.6637°W
**1693**	USA: Virginia: Danville City Co., Danville, Dan River, 36.5828°N, 79.4246°W
**1694**	USA: Iowa: Hamilton Co., Boone River near Stratford, 275 m, 42.3123°N, 93.9327°W
**2343**	USA: Texas: Bastrop Co., Colorado River near Utley, 115 m, 30.1853°N, 97.4496°W
***Bembidion mimbres* sp. nov.**	
**0280**	USA: New Mexico: Grant Co., Gila River near Gila, 1370 m 32.969°N, 108.587°W
**1220**	USA: New Mexico: Grant Co., Billings Vista, Gila River, 1310 m 32.8137°N, 108.6031°W
**1267**	USA: New Mexico: Grant Co., Billings Vista, Gila River, 1310 m 32.8137°N, 108.6031°W
**1944**	USA: New Mexico: Grant Co., Billings Vista, Gila River, 1310 m, 32.8137°N, 108.6031°W
**2117**	USA: New Mexico: Grant Co., Gila River, Billings Vista, 1320 m, 32.8163°N, 108.6032°W
**2118**	USA: New Mexico: Grant Co., Gila River, Billings Vista, 1320 m, 32.8163°N, 108.6032°W
**2119**	USA: New Mexico: Grant Co., Gila River, Billings Vista, 1320 m, 32.8163°N, 108.6032°W
**2131**	USA: New Mexico: Grant Co., Gila River, Billings Vista, 1320 m, 32.8163°N, 108.6032°W
**2134**	USA: New Mexico: Grant Co., Gila River, Billings Vista, 1320 m, 32.8163°N, 108.6032°W
**2135**	USA: New Mexico: Grant Co., Gila River, Billings Vista, 1320 m, 32.8163°N, 108.6032°W
***Bembidion transversale* Dejean**	
**2157**	USA: Wyoming: Albany Co., Laramie River, Laramie, 2175 m, 41.2897°N, 105.6224°W
**2160**	Canada: Nova Scotia: Hantsport, Halfway River, 45.0487°N, 64.1835°W
**2486**	USA: Colorado: Fremont Co., Arkansas River at Texas Creek, 1880 m, 38.4106°N, 105.5844°W
**4219**	USA: Oregon: Harney County, Banks of Silver Creek, 1379 m, 43.7278°N, 119.6256°W
**4690**	USA: Montana: Gallatin Co., Beaver Creek along Hwy 287, 1969 m, 44.8633°N, 111.3679°W
**4927**	USA: Montana: Gallatin Co., Beaver Creek along Hwy 287, 1969 m, 44.8633°N, 111.3679°W
**5064**	USA: Washington: Whitman Co., Palouse River 6 mi NE Colfax, 600 m 46.9259°N, 117.3037°W
**5612**	USA: Oregon: Wallowa Co., Wallowa Lake State Park, 1334 m 45.2841°N, 117.2075°W
**5613**	USA: Oregon: Harney Co., Banks of Donner und Blitzen River 1296 m, N 42.8002, W 118.8682
***Bembidion erosum* Motschulsky**	
**2162**	Canada: British Columbia: Hope, Fraser River near mouth of Coquihalla River, 49.3961°N, 121.4351°W
**2194**	Canada: British Columbia: Hope, Fraser River near mouth of Coquihalla River, 49.3961°N, 121.4351°W
**2596**	USA: California: Del Norte Co. rt 101 @ Wilson Creek, 41.60530°N, 124.10060°W
**2607**	USA: California: Del Norte Co. rt 101 @ Wilson Creek, 41.60530°N, 124.10060°W
**3561**	USA: California: Del Norte Co., Wilson Creek, 3 m, 41.6051°N, 124.1005°W
**3562**	USA: California: Del Norte Co., Wilson Creek, 3 m, 41.6051°N, 124.1005°W
**3584**	USA: California: Del Norte Co., Wilson Creek, 3 m, 41.6051°N, 124.1005°W
**4033**	USA: Oregon: Curry Co., Floras Creek at route 124 SE Langlois, 21 m, 42.9132°N, 124.4251°W
**4050**	USA: California: Monterey Co., Big Sur River, Andrew Molera State Park, 7 m, 36.285°N, 121.8544°W
**4212**	USA: California: Del Norte Co., Wilson Creek, 3 m, 41.6051°N, 124.1005°W
***Bembidion corgenoma* sp. nov.**	
**2165**	USA: Washington: Whatcom Co., Nooksack River 1.4 mi S of Deming, 70 m, 48.808°N, 122.2019°W
**2180**	USA: California: Sonoma Co., Russian River, 3 mi NE Healdsburg
**2181**	USA: California: Marin Co., Nicasio Reservoir, 70 m, 38.088°N, 122.7383°W
**2190**	Canada: British Columbia: Clearwater, N. Thompson River, 440 m, 51.6395°N, 120.0294°W
**2346**	USA: Nevada: Eureka Co., I-80W bridge 1.6 mi E exit 254 (Dunphy), Humbolt R., 1408 m 40°42.31‘N, 116°31.87‘W
**2597**	USA: California: Del Norte Co. rt 101 @ Wilson Creek, 41.60530°N, 124.10060°W
**2608**	USA: California: Del Norte Co. rt 101 @ Wilson Creek, 41.60530°N, 124.10060°W
**2973**	USA: Oregon: Benton Co., Corvallis, Willamette River, 60 m, 44.5491°N, 123.2451°W
**3021**	USA: Idaho: Cassia Co., Sublett Res.
**3205**	USA: Oregon: Benton Co., Corvallis, Willamette River, 60 m, 44.5491°N, 123.2451°W
**3559**	USA: California: Del Norte Co., Wilson Creek, 3 m, 41.6051°N, 124.1005°W
**3560**	USA: California: Del Norte Co., Wilson Creek, 3 m, 41.6051°N, 124.1005°W
**3583**	USA: California: Del Norte Co., Wilson Creek, 3 m, 41.6051°N, 124.1005°W
**3772**	USA: California: Tehama Co., Red Bluff, Sacramento River, 73 m, 40.1759°N, 122.229°W
**4032**	USA: Oregon: Coos Co., Crooked Creek S of Bandon, 7 m, 43.0814°N, 124.4335°W
**4034**	USA: Oregon: Curry Co., Floras Creek at route 124 SE Langlois, 21 m, 42.9132°N, 124.4251°W
**4052**	USA: California: Monterey Co., Big Sur River, Andrew Molera State Park, 7 m, 36.285°N, 121.8544°W
**4054**	USA: California: San Luis Obispo Co., Pismo State Beach, 4 m, 35.0999°N, 120.6267°W, 29.iv.2014
**4218**	USA: California: San Luis Obispo Co., San Simeon State Park, San Simeon Creek, 4 m, 35.5955°N, 121.1233°W
**4959**	USA: Nevada: Carson City Co., Carson River at Silver Saddle Ranch, SE Carson City, 1405 m 39.1315°N, 119.706°W
**4961**	USA: California: Tehama Co., Red Bluff, Sacramento River, 73 m 40.1759°N, 122.229°W
**4962**	USA: California: Tehama Co., Red Bluff, Sacramento River, 73 m 40.1759°N, 122.229°W
**5065**	USA: Oregon: Linn Co., Willamette River, Truax Island, 60 m 44.5853°N, 123.1913°W
**5670**	USA: Oregon: Benton Co., Corvallis, Willamette River, 60 m 44.5491°N, 123.2449°W
**5671**	USA: Oregon: Benton Co., Corvallis, Willamette River, 60 m 44.5491°N, 123.2449°W
**5672**	USA: Oregon: Benton Co., Corvallis, Willamette River, 60 m 44.5491°N, 123.2449°W
**5673**	USA: Oregon: Benton Co., Corvallis, Willamette River, 60 m 44.5491°N, 123.2449°W

#### Sequence alignment

Alignment was not difficult for any of the protein-coding genes. There were no insertion or deletions (indels) evident in the sampled CAD, ArgK, Topo, *wg*, or COI sequences. An alignment of 28S was conducted in MAFFT version 7.130b (Katoh and Standley 2013), using the L-INS-i search option and otherwise default parameter values.

#### Molecular phylogenetic analysis

Maximum likelihood analysis was conducted for each gene individually using IQ-TREE version 1.6.7.1 (Nguyen et al. 2015), as orchestrated by Mesquite’s Zephyr package (Maddison and Maddison 2018b, c). The ModelFinder feature within IQ-TREE (Kalyaanamoorthy et al. 2017) was used to find the optimal character evolution models. The MFP model option was used for 28S, and the TESTMERGE option for the protein-coding genes. The TESTMERGE option sought the optimal partition of sites, beginning with the codon positions in different parts. Twenty searches were conducted for the maximum-likelihood tree for each matrix.

For the *B. transversale* group, a multi-species coalescent approach was conducted with the 28S, COI, CAD, and Topo data to provide an algorithmic analysis of species boundaries. STACEY version 1.2.5 (Jones 2017) was used as implemented in BEAST version 2.6.2 (Bouckaert et al. 2014), with the epsilon value set to 1*10–4, CollapseWeight parameters to 0.5 and 10, and with a Beta prior. I evaluated sampling sufficiency using ESS values in Tracer version 1.7.1 (Rambaut et al. 2018); after four independent runs of 1E9 generations each, all ESS values exceeded 200, except for mutationRate.s:Topo, whose ESS value was 191. As I saved trees every 100,000 generations, with the first 10% of the trees discarded as the burn-in period, this yielded a sample of 72,000 trees.

## Data resources

Sequences have been deposited in GenBank with accession numbers MW151366–MW151563. Aligned data for each specimen as well as files containing inferred trees for each gene are available in Supplementary material S1 and S2, and have been deposited in the Dryad Digital Repository, https://doi.org/10.5061/dryad.18931zcw1.

## Results

### Molecular results

**Figure 5.**
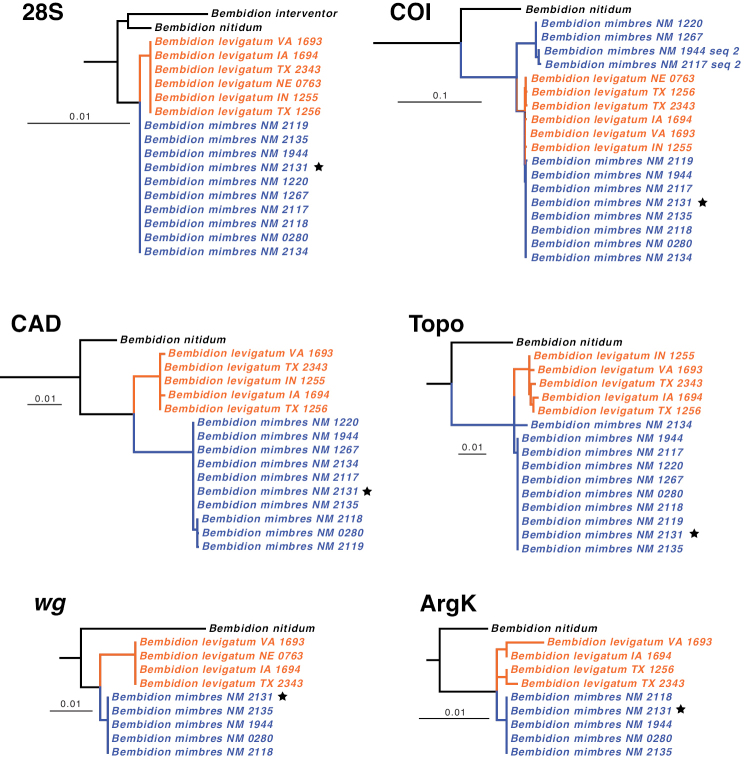
Maximum likelihood gene trees of subgenus Hydrium. Only *B. levigatum* + *B. mimbres* + its sister group shown (other taxa were present in the analysis, and reconstructed outside this clade, but were removed after the analysis to simplify this figure). Holotype of *B. mimbres* indicated by a star. Scale bar 0.1 units, as reconstructed by IQ-TREE.

In the analysis of DNA data for the subgenus Hydrium, *B. levigatum* and *B. mimbres* sp. nov. differed in all genes except COI (Fig. 5), providing evidence that they are two separate species. In all gene trees except 28S, *B. nitidum* was the sister group of *B. levigatum* +*B. mimbres* (Suppl. materials 1).

The majority of specimens of *B. levigatum* and *B. mimbres* were indistinguishable in COI, but there were four sequences of *B. mimbres* that formed a separate clade (for specimens 1220, 1267, as well as the second sequences of 1944 and 2117). These four sequences have 29 sites at which they differ from all other sampled *B. levigatum* + *B. mimbres*, at 20 of which these four sequences have the same base as in at least one other sampled *Hydrium* species. These four sequences might be nuclear copies or numts (Thalmann et al. 2004), or they could represent the effects of *Wolbachia* infections (Smith et al. 2012). Although it is possible these are the true mitochondrial copies of COI, and that the other sequences are numts, the evidence points to the four unusual sequences being something other than true mitochondrial copies: the chromatograms for these four unusual sequences have several double peaks, indicating polymorphism within the PCR products for non-synonymous differences. These four sequences have been deposited in GenBank as “COI-like” sequences.

**Figure 6.**
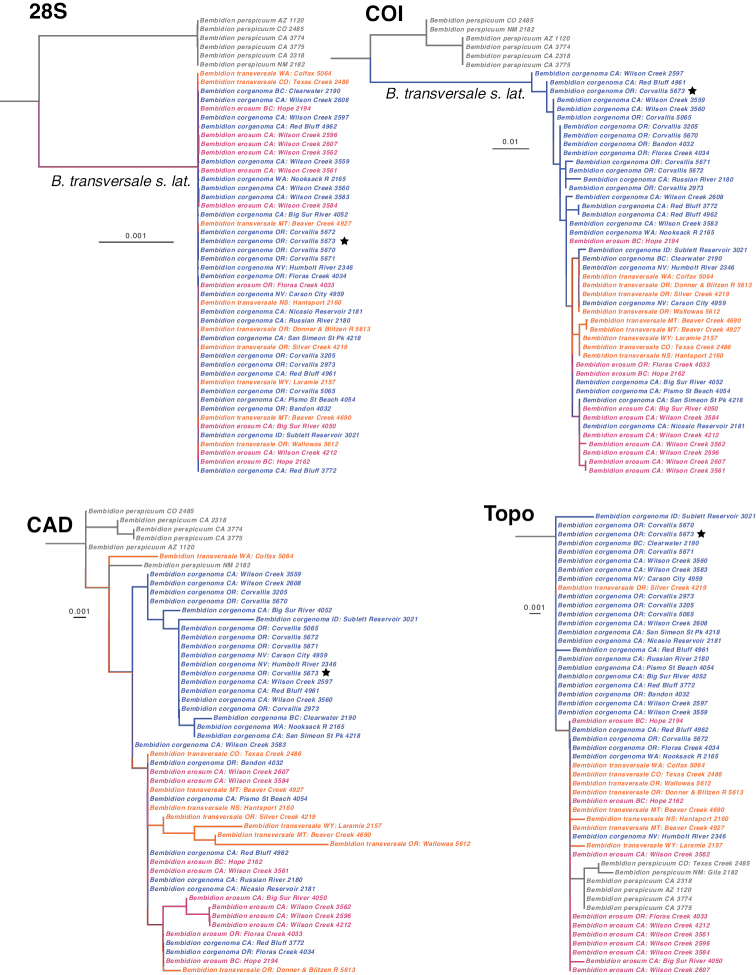
Maximum likelihood gene trees of the *Bembidion transversale* subgroup. *B. mexicanum* subgroup and *B. sarpedon* were present in the analysis, and reconstructed outside this clade, but were graphically removed to simplify this figure. Holotype of *B. corgenoma* indicated by a star. Scale bar as reconstructed by IQ-TREE.

**Figure 7.**
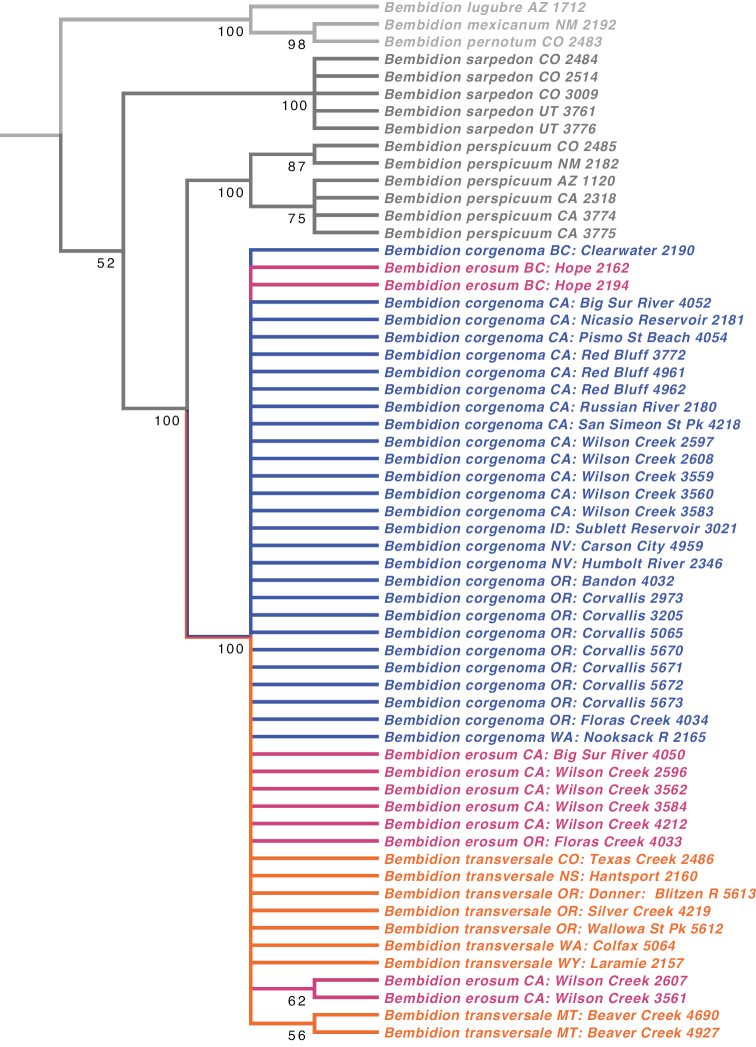
Majority rule consensus tree of trees found from a STACEY analysis. Numbers on branches are estimates of the Bayesian posterior probability of a clade, expressed as a percentage.

In each of the four genes studied in the *B. transversale* group, the maximum likelihood tree showed a monophyletic *B. transversale* subgroup (Suppl. materials 2), with *B. perspicuum* and *B. transversale* s. l. (= *B. transversale* + *B. erosum* + *B. corgenoma*) forming a clade, the sister of which is *B. sarpedon*. None of the three species within *B. transversale* s. l. form a clade in any of the four genes studied (Fig. 6). The multi-species coalescent STACEY tree also showed no distinction between these species based upon the combined analysis of 28S+COI+CAD+Topo (Fig. 7).

### Cytogenetic results

All males examined are inferred to have 22 autosomes (i.e., 11 pairs of autosomes) and an XY/XX sex chromosome system (Table 4).

Re-examination of voucher specimens identified as *B. transversale* in Maddison (1985) showed that they belong to three species. The specimens from Alberta and near Fernie, BC, are *B. transversale*; the specimen from near Cache Creek, BC, is *B. corgenoma*; the specimen from Salmon Valley, BC, is *B. erosum*. The specimens called “*B.* sp nr *transversale*-Nr 1” are *B. pernotum*, and the specimens called “*B.* sp nr *transversale*-Nr 2” are *B. lugubre*. The specimens reported as belonging to “*B.* sp. nr. *transversale*” in Pflug et al. (2020) are all *B. corgenoma*. These new identifications are incorporated into the summary shown in Table 4.

**Table 4.** Chromosome numbers and sex chromosomes of Bembidion (Hydrium) and *Bembidion transversale* group males. The Sample column indicates the total number of specimens examined in this paper and in previous papers. “1” in Reference indicates Maddison (1985); “2” indicates Pflug et al. (2020).

	2n male	Sample	Locality	Reference
*B. levigatum*	22+XY	2♂	TX	1
*B. mimbres*	22+XY	1♂	NM	this paper
*B. transversale*	22+XY	5♂2♀	AB, BC, CO	1, this paper
*B. erosum*	22+XY	4♂	BC, OR	1, this paper
*B. corgenoma*	22+XY	15♂	OR, BC	1, 2
*B. perspicuum*	22+XY	4♂	CO, AZ	1, this paper
*B. sarpedon*	22+XY	3♂	CO	this paper
*B. pernotum*	22+XY	6♂	CO	1, this paper
*B. mexicanum*	22+XY	5♂	CO, AZ	1, this paper
*B. lugubre*	22+XY	11♂	AZ, CA, Mexico	1, this paper

### Morphological results

Morphological results for Bembidion (Hydrium) are presented in the taxonomic section below.

Members of the *B. transversale* subgroup are very similar morphologically. DNA sequence data of 28S, COI, CAD, and Topo do not reveal any consistent phylogenetic structure within *B. transversale* s. l. (Figs 6, 7), suggesting that it is perhaps a single species. In all other bembidiines investigated to date (e.g., Maddison 2008; Maddison and Cooper 2014; Maddison and Sproul 2020; Sproul and Maddison 2017), every form judged by morphological evidence as a distinct species is revealed as a clade in the tree of at least one of these four genes. I detected no variation in chromosomes within the group (Table 4); although *Bembidion* species typically have similar chromosomes (Maddison 1985), some subgenera have species that differ by chromosome number (Maddison 2008). However, in spite of the lack of genetic evidence supporting multiple species, the morphological results reveal that this complex consists of at least three distinct but very similar species.

Examination of primary types (documented in the Taxonomic Treatment section, below) indicates that two of the species have names (*Bembidion transversale* and *B. erosum*), and the third is described here as *B. corgenoma*; these names will be used in advance of the Taxonomic Treatment to simplify the text.

The morphological evidence indicating that there are three species includes color (Figs 11A–C, 12), mentum shape (Fig. 13), and male genitalic structure (Figs 14–17), with the patterns summarized below and graphically in Fig. 18.

The dark and pale western species (*B. erosum* and *B. corgenoma*) are broadly sympatric from southern California through British Columbia (Figs 19, 20), and are found microsympatrically (on the same gravel bank) at Wilson Creek, Del Norte County, California (41.6051°N, 124.1005°W), as well as along Floras Creek, Curry County, Oregon (42.9132°N, 124.4251°W), and the Siletz River E of Kernville, Oregon (44.8720°N, 123.9223°W). They differ consistently in size of a sclerotized lobe of the internal sac (Fig. 15B vs. C); the thickness of the tip of the flagellar sheath, with *B. corgenoma* having a somewhat triangular sclerotized region of the tip (arrow in Fig. 16E), as opposed to a thin dark line in *B. erosum* (Fig. 16C, D); and color, with *B. erosum* being generally darker (Fig. 11B) than *B. corgenoma* (Fig. 11C), especially the appendages (Fig. 12B vs C). In addition, most males of *B. erosum* have the ventral surface of the apex of the aedeagus more strongly curved downward (Fig. 14C, D). Although there are no universal distinctions between the two species in the genes sequenced, the six *B. erosum* and five *B. corgenoma* sequenced from the gravel bank shown in Fig. 4B consistently differ in one base in Topoisomerase, suggesting, combined with differences in genitalia and color, that there is no or extremely limited gene flow at that locality.

The ranges of the two generally paler species (*B. transversale* and *B. corgenoma*) overlap in Nevada, Washington, Idaho, and Montana (Fig. 19), and there are three localities at which they co-occur (16 mi W Lolo Pass, Ravalli Co., Montana; Walla Walla, Washington; Spokane, Washington; all in OSAC). There are subtle but consistent differences in the male genitalia, as well as striking differences in the mentum, and I am convinced any gene exchange in the region of overlap is minimal. In the overlap region there are a very few specimens of *B. transversale* with paler antennae (similar to typical *B. corgenoma*), and there is one population at Hayden Lake, Idaho (CAS), which contains *B. transversale* typical in all regards except for one specimen that has the anterior margin of the mentum somewhat intermediate between the two species (Fig. 13B). With these minor exceptions, the differences in mentum, genitalia, and color are consistent throughout the overlap range among the many males whose genitalia were dissected.

*Bembidion erosum* and *B. transversale* are the two most similar species within the trio, differing most notably in the anterior lateral region of the mentum: in *B. erosum* this region is large and triangular, similar to that standard in *Bembidion* (i.e., like those shown in Fig. 13A), in contrast to the modified mentum of *B. transversale* (Fig. 13B–D), in which the anterior lateral region is much reduced. The male genitalia are very similar, both having a larger lobe on the basal sclerite (Fig. 15A, B), and a thin, non-triangular apex to the flagellar sheath (Fig. 16A–D). However, the flagellar sheath is more dorso-ventrally compressed in *B. transversale* (Fig. 16A, B), and the flagellar complex is thinner (arrow in Fig. 17A, B). With one exception, the known ranges of *B. erosum* and *B. transversale* do not overlap, with *B. erosum* in the United States being restricted to the Cascades and west, and *B. transversale* only known from east of the Cascades (Figs 19, 20). The one exception is in eastern Washington: there is a series of seven specimens of *B. erosum* labeled “Colbert, Wash / V 30 1937 / Dan Bonnell”. The two males from this series have typical *B. erosum* genitalia, and the mentum has the anterior lateral region large and triangular. Colbert is less than 20 km NNE of Spokane, where both *B. transversale* and *B. corgenoma* have been collected. As the only record of *B. erosum* east of the Cascades, some doubt is cast upon its authenticity, but there is no reason otherwise to question the locality data.

In addition, there is a form in the Sierra Nevada of California that requires further examination. My limited study of it indicates that it has all of the morphological features of *B. transversale*, except that it has a normal mentum shape, with large and triangular anterior lateral regions.

## Taxonomic Treatment

### Subgenus Hydrium

The subgenus Hydrium, as defined by Maddison (2012), contains seven species in the Palaearctic region (Marggi et al. 2017) as well as five species in the Nearctic region (Lindroth 1963): *Bembidion nitidum*, *B. interventor*, *B. obliquulum*, *B. levigatum*, and the new species described here.

The species key in Lindroth (1963) can be modified as follows to take into account the new species.

**Table:** 

19	More than two setae on the clypeus, and at least one long seta on the front angle of the prothorax. Elytra without the typical pair of distinguishable dorsal punctures but most intervals with a row of small punctures, each carrying a long seta	**19A**
–	Clypeus with only two setae. Elytra with two dorsal punctures on third interval, otherwise without setigerous punctures	**20**
19A	Elytra with a row of long setae on all intervals; prothorax wide, sides very rounded (Fig. 8A)	***B. levigatum***
–	Elytra with a row of long setae on most intervals, but lacking on intervals 2 and 4. Prothorax narrower, with straighter sides (Fig. 8B)	***B. mimbres***

**Figure 8.**
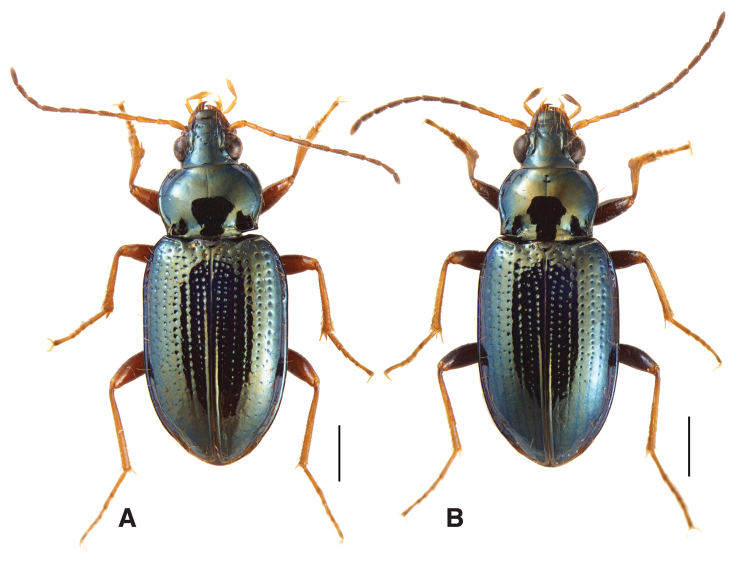
Adult males of subgenus Hydrium**A***Bembidion levigatum* (voucher V100820, from USA: Utah: Grand Co., Moab, Colorado River, 1190 m, 38.5739°N, 109.5769°W) and **B***B. mimbres* (a paratype from the type locality, voucher V101458). Scale bars: 1 mm.

#### Bembidion mimbres sp. nov.

Taxon classificationAnimaliaColeopteraCarabidae

FC03C6EA-A73F-53A4-83C0-BACEB1EEEF83

http://zoobank.org/5F95B4F6-9FFD-4841-8E69-794A258381A2

##### Holotype.

Male, in OSAC, labeled: “USA: New Mexico: Grant Co., Gila River, Billings Vista, 1320 m, 32.8163°N, 108.6032°W, 11.viii.2005. DRM 05.043. D.R. & J.H.A. Maddison”, “David R. Maddison DNA2131 DNA Voucher” [pale green paper], “HOLOTYPE Bembidion mimbres David R. Maddison” [partly handwritten, on red paper], “Oregon State Arthropod Collection OSAC_0002000007 [matrix code]” [printed on both sides of white paper]. Genitalia in glycerin vial pinned beneath specimen; extracted DNA stored separately. GenBank accession numbers for DNA sequences of the holotype are MW151386, MW151400, MW151414, MW151425, MW151432, and MW151443.

##### Paratypes

**(116).** USA: New Mexico: Grant Co., Gila River, Billings Vista, 1320 m, 32.8163°N, 108.6032°W [Type locality] (44: OSAC, USNM, MCZ, NHMUK, MNHM, MSBA), USA: New Mexico: Grant Co., Billings Vista, Gila River, 1310 m, 32.8137°N, 108.6031°W (28: OSAC, CAS, UAIC, EMEC); USA: New Mexico: Grant Co., Gila River near Cliff, 1350 m, 32.9124°N, 108.5897°W (12: OSAC); USA: New Mexico: Grant Co., Gila River near Gila, 1370 m, 32.9692°N, 108.5868°W (3: OSAC); USA: New Mexico: Grant Co., Gila River near Gila, 1370 m, 32.969°N, 108.587°W (4: OSAC); USA: New Mexico: Grant Co., Gila River, Gila National Forest, 1315 m, 32.8167°N, 108.6035°W (14: OSAC); USA: New Mexico: Gila R., jct US 260, nr. Gila (1: UASM); USA: Arizona: Navajo Co., Carrizo Ck nr. Carrizo (10: UASM).

##### Type locality.

USA: New Mexico: Grant Co., Gila River, Billings Vista, 32.8163°N, 108.6032°W.

##### Derivation of specific epithet.

*Bembidion mimbres* is named in honor of the people of the Mimbres culture, who lived alongside this species, including at the type locality, and who illustrated the insects in their world on their pottery (Hegmon, et al. 2018). The name is to be treated as a noun in apposition.

##### Diagnosis and description.

**Figure 9.**
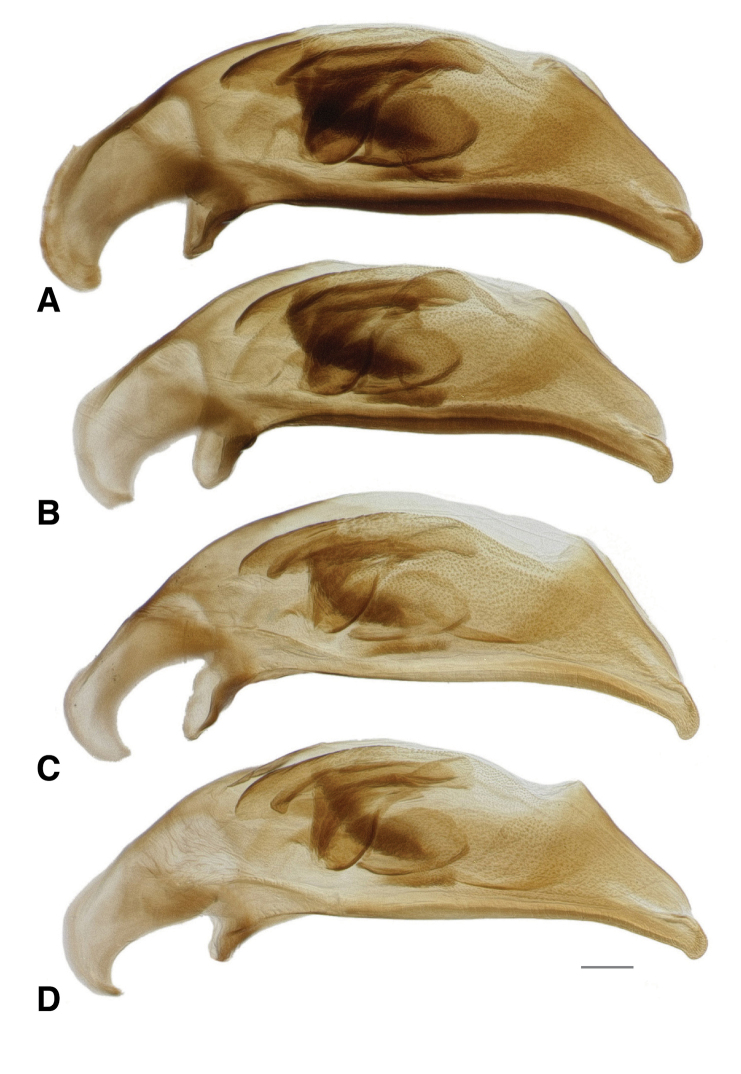
Male genitalia of subgenus Hydrium**A***B. levigatum* (voucher DNA1693, Virginia: Danville City Co., Danville) **B***B. levigatum* (voucher DNA2343, Texas: Bastrop Co., Colorado River near Utley) **C***B. mimbres* (voucher DNA2134, USA: New Mexico: Grant Co., Gila River, Billings Vista) **D***B. mimbres* (voucher DNA2135, USA: New Mexico: Grant Co., Gila River, Billings Vista). Scale bar: 0.1 mm.

Adults of this species are relatively large *Bembidion* (5.3–6.3 mm in length), with a striking appearance because of the smooth and shiny dorsal surface with its metallic reflections (Figs 1, 8B). Body piceous, with an aeneous, green, or blue metallic reflection. Legs with tarsi and tibiae testaceous, femora infuscated. First three antennomeres testaceous, with the tip of the third infuscated in some specimens; fourth basally testaceous. Palps testaceous except for the penultimate maxillary article, which is infuscated. Mentum with anterior lateral regions large and triangular as typical for a *Bembidion*; mentum tooth incised at tip, and thus bifid (similar to *B. levigatum*). Prothorax with relatively straight sides, especially posteriorly (Fig. 8B); with distinct posterolateral carina close to the lateral margin; posterior region of pronotum smooth, impunctate. Lateral bead of elytra extending inside shoulder well toward the midline, sharply angulate. Striae consisting of rows of distinct punctures, without an associated groove; on stria 1 complete; striae 2–6 absent in the posterior third; stria 7 virtually absent, represented by at most minute punctulae. Dorsal surface lacking microsculpture, and thus very shiny. More than two setae on the clypeus, and extra setae on the frons, at least anteriorly; at least one long seta near the front angle of the prothorax; prosternum with at most two setae. Elytra without the typical pair of distinguishable setose punctures associated with interval 3, but with a row of long setae on all intervals except 2 and 4. Aedeagus (Fig. 9C,D) with ventral margin thinner, and with internal sac sclerites very similar to those of *B. levigatum*, but with slight differences, especially basally.

Most easily distinguished from *B. levigatum* by the narrower prothorax with straighter sides (Fig. 8B), the lack of setae on elytral intervals 2 and 4, and having at most one or two setae on the prosternum.

##### Additional characteristics.

Diploid chromosome number 24, with 11 pairs of autosomes and an XY/XX sex chromosome system.

##### Geographic distribution.

**Figure 10.**
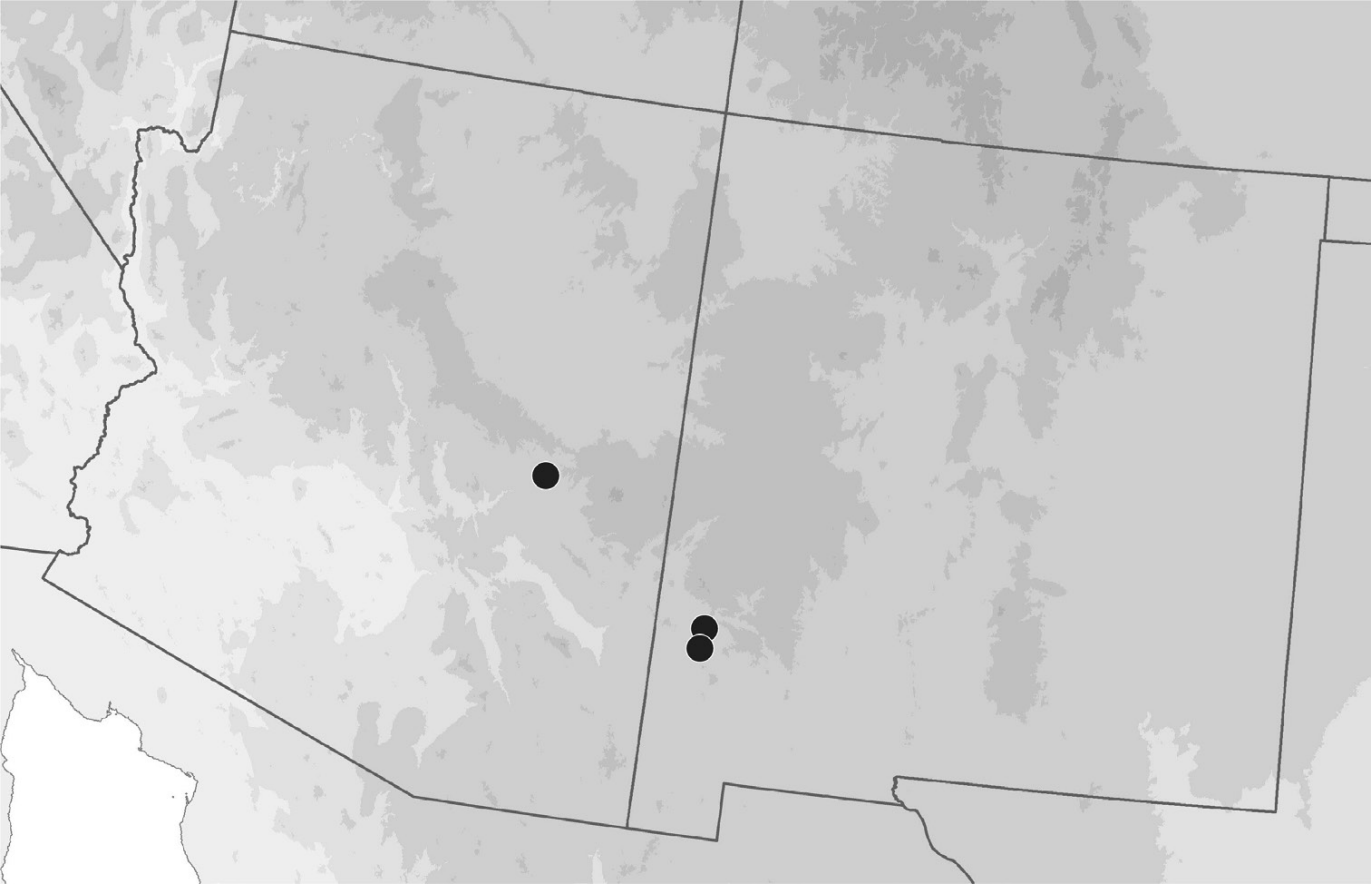
Geographic distribution of *B. mimbres* in Arizona and New Mexico.

Known from the Gila River watershed in Arizona and New Mexico (Fig. 10).

##### Habitat.

At the type locality, found at night on damp sandy soil about 2–4 meters from the river shore, in the shade of a large willow (Fig. 2). At a site a few meters away, 28 specimens were found at night on damp clay/sand soil among small *Salix* and *Populus* saplings 2–8 meters from the water’s edge; in spite of extensive searching in the same area, only one specimen was found during the day. At other sites along the Gila River, found in habitats similar to those in which *B. levigatum* is found, on damp sand and silt on the steep upper bank of the river, mostly at night.

#### Bembidion levigatum

Taxon classificationAnimaliaColeopteraCarabidae

Say

33DA63FC-6AE4-5AAE-A9DC-9E2C4B5B2184

Bembidium levigatum Say, 1823: 84. Lectotype female in MNHN, designated by Lindroth and Freitag (1969). Type locality Missouri.
Bembidion laevigatum delawarense Casey, 1924: 24. Holotype male in USNM (type number 36814), examined. Type locality Pennsylvania.

##### Diagnosis and geographic distribution.

Adults of this species are large and distinctively wide, with a broad, rounded prothorax (Fig. 8A), and with a very shiny dorsal surface, with a green or bluish metallic reflection. The clypeus, frons, anterior corner of the prothorax, and all elytral intervals have long setae in addition to the standard set in *Bembidion*; the prosternum has four or more setae. Aedeagus as in Fig. 9A, B. A very widespread species, found throughout most of the eastern United States and a small region of southern Canada, from Maine to Florida, north and west to Alberta and Montana, south to Utah, New Mexico, Texas, and Mexico (Bousquet 2012). I have also seen specimens from the Grand Canyon in northern Arizona (two specimens in MSBA labeled “USA AZ Coconino Co Grand Canyon Nat. Park, N36.77 W111.655 RMBL 29-30 August 2002 coll. Cobb, Brantley, Lightfoot”).

### *Bembidion transversale* species group

The *Bembidion transversale* group contains large *Bembidion* found primarily on river shores of cobble, gravel, and sand from Canada to Guatemala. Members of the group are characterized by large size (5.8–8 mm); posterolateral carina of pronotum lacking or indistinct and somewhat oblique; lateral bead of elytra not prolonged onto shoulder; crista clavicularis absent; elytral striae distinct and mostly complete; elytral microsculpture transverse; two discal setae of elytra in or close to third stria. It belongs to what has been called the Nearctic *Ocydromus* Clade (Maddison 2012), although that group is not closely related to subgenus Ocydromus. The only subgeneric name available for the Nearctic *Ocydromus* Clade is *Leuchydrium* Casey, although the type species (*Bembidion tigrinum* LeConte) is quite distant from the *B. transversale* group (Maddison 2012).

There are now eight recognized species in the *B. transversale* group in the United States and Canada:

*Bembidion transversale* subgroup

*Bembidion transversale* Dejean, 1831

*Bembidion erosum* (Motschulsky, 1850)

*Bembidion corgenoma* Maddison, sp. nov.

*Bembidion perspicuum* (LeConte, 1848)

*Bembidion sarpedon* Casey, 1918

*Bembidion mexicanum* subgroup

*Bembidion mexicanum* Dejean, 1831

*Bembidion lugubre* LeConte, 1857

*Bembidion pernotum* Casey, 1918

There is a total of 20 species-group names that have been applied to members of the *B. transversale* group (for details beyond those provided below, see Maddison and Swanson (2010)). I have examined detailed photographs of the primary type of one (the holotype of *Bembidium transversale* Dejean, in the MNHN), and the primary types themselves of 18. The twentieth lacked a type series, and a neotype is designated below.

The species key in Lindroth (1963) can be modified as follows to take into account species in the *B. transversale* subgroup. Specimens from this group are not easy to identify.

**Table:** 

145	Prothorax (Lindroth 1963: figs 168a–b) without or with very faint, oblique latero-basal carina	**145A**
–	Prothorax with latero-basal carina well developed, less oblique	**146**
145A	Mentum with anterior lateral region reduced, not triangular, each consisting of a mesal denticle and a more lateral rounded bump (Fig. 13B–D). Antenna with at least the second and third antennomeres apically infuscated. Tip of aedeagus not abruptly curved downward (Fig. 14A, B); basal sclerotized lobe large (Fig. 15A); apex of flagellar sheath with long, thin dark line (Fig. 16A, B)	***B. transversale***
–	Mentum with anterior lateral region as typical for a *Bembidion*: triangular, large, and with anterior margin significantly anteriad of the central tooth (Fig. 13A). Other characteristics either as mentioned above or not	**145B**
145B	Paler, with antennae gradually becoming slightly darker toward the apex; pronotum in most specimens dark rufous. Prothorax with lateral margins more rounded, very shiny, with weaker microsculpture and less punctuation. Relatively flat elytral intervals with small punctures in striae. Aedeagus with ventral margin having a slight downward bulge. Internal sac sclerite complex of male genitalia narrower in side view, with relatively long and thin flagellar complex. Known from NM, CO, WY, UT, AZ	***B. sarpedon***
–	Darker, with at least antennomeres 4–11 infuscated; pronotum rufous or piceous. Prothorax with lateral margins less rounded, less shiny, and in most specimens with more punctures. Aedeagus without ventral bulge. Internal sac sclerite complex less narrow, with a dorso-ventrally wider flagellar complex	**145C**
145C	Prothorax with later margins less sinuate, with more notable punctures in the basal region (Maddison and Swanson 2010: fig. 4B); aedeagus with ostial flag more dorsal, and with a more abrupt curve at its anterior end (Maddison and Swanson 2010: fig. 6B)	***B. perspicuum***
–	Prothorax with lateral margins more sinuate, flatter, with a smoother basal region (Maddison and Swanson 2010: fig. 4A); aedeagus with ostial flag extending further ventrally, and with gentler curvature (Maddison and Swanson 2010: fig. 6A)	**145D**
145D	Legs and basal three antennomeres pale, testaceous or rufo-testaceous. Elytral striae deeper. Tip of aedeagus not abruptly curved downward (Fig. 14E, F); basal sclerotized lobe small (Fig. 15C); apex of flagellar sheath with dark area triangular (Fig. 16E, F)	***B. corgenoma***
–	Legs in most specimens darker (in southern specimens infuscated); second and third antennomeres infuscated, at least apically. Elytral striae shallower. Tip of aedeagus abruptly curved downward (Fig. 14C, D); basal sclerotized lobe large (Fig. 15B); apex of flagellar sheath with long, thin dark line (Fig. 16C, D)	***B. erosum***

#### Bembidion transversale

Taxon classificationAnimaliaColeopteraCarabidae

Dejean, 1831

CEB92433-FB0A-51B2-9721-3AC6F6FF624C

Bembidium transversale Dejean, 1831:110. Holotype female, in MNHN, examined by Kipling Will, who provided photographs that confirmed the identification. Type locality restricted to Nipigon, Ontario, by Lindroth (1963).

##### Diagnosis and geographic distribution.

**Figure 11.**
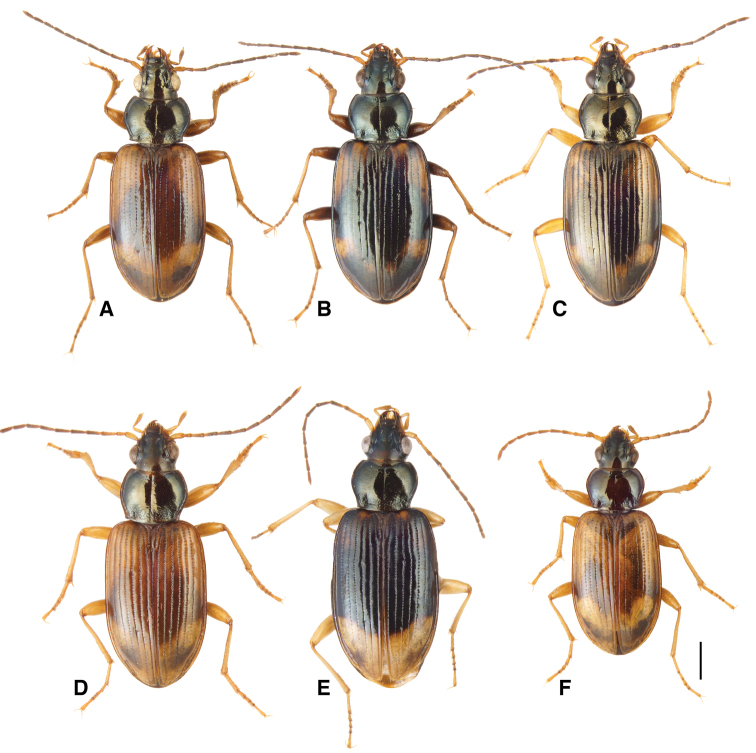
Adult males of *Bembidion transversale* subgroup members **A***B. transversale* (voucher V101454, Canada: Alberta: Burbank, junction of Red Deer and Blindman Rivers, 52.3542°N, 113.7556°W) **B***B. erosum* (voucher V101453, USA: California: Del Norte Co., Wilson Creek, 3 m, 41.6051°N, 124.1005°W). **C***B. corgenoma* (voucher V101452, from type locality) **D***B. perspicuum*, light form (voucher V101461, USA: Arizona: Cochise Co., San Pedro R at Charleston, 31.6239°N, 110.1722°W) **E***B. perspicuum*, dark form (neotype of *Bembidium haplogonum* Chaudoir, USA: California: Lake Co., North Branch Cache Creek at hwy 20, 305 m 38.9881°N, 122.5400°W) **F***B. sarpedon* (voucher V101459, USA: Colorado: Las Animas Co., Cokedale, Reilly Canyon, 37.1346°N, 104.6114°W). Scale bar 1.0 mm.

**Figure 12.**
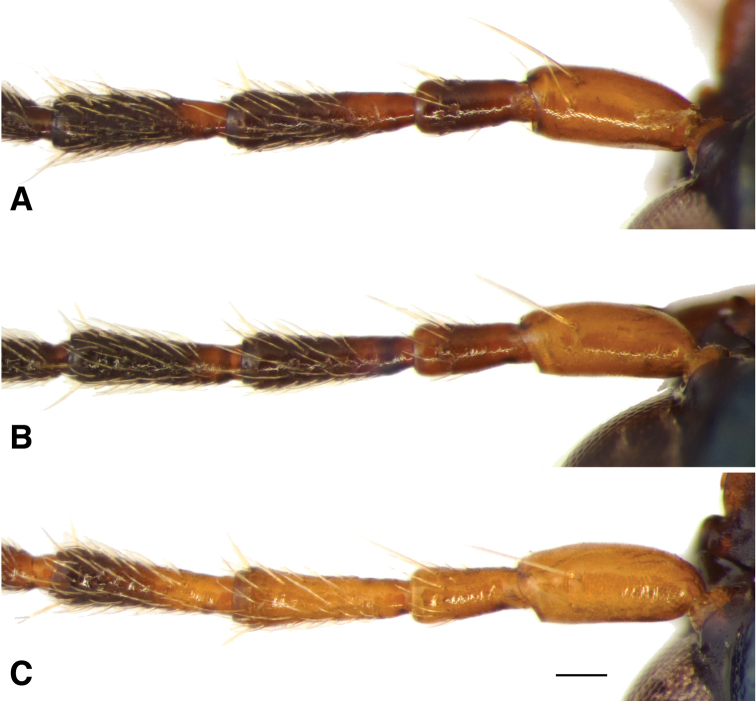
Antennae of *B. transversale* subgroup **A***B. transversale* (voucher V101457, Canada: Ontario: Thunder Bay Dist., Rossport) **B***B. erosum* (voucher V101456, USA: California: Del Norte Co., Wilson Creek, 3 m, 41.6051°N, 124.1005°W) **C***B. corgenoma* (voucher V101455, USA: Oregon: Coos Co., Crooked Creek S of Bandon, 43.0814°N, 124.4335°W). Scale bar 0.1 mm.

**Figure 13.**
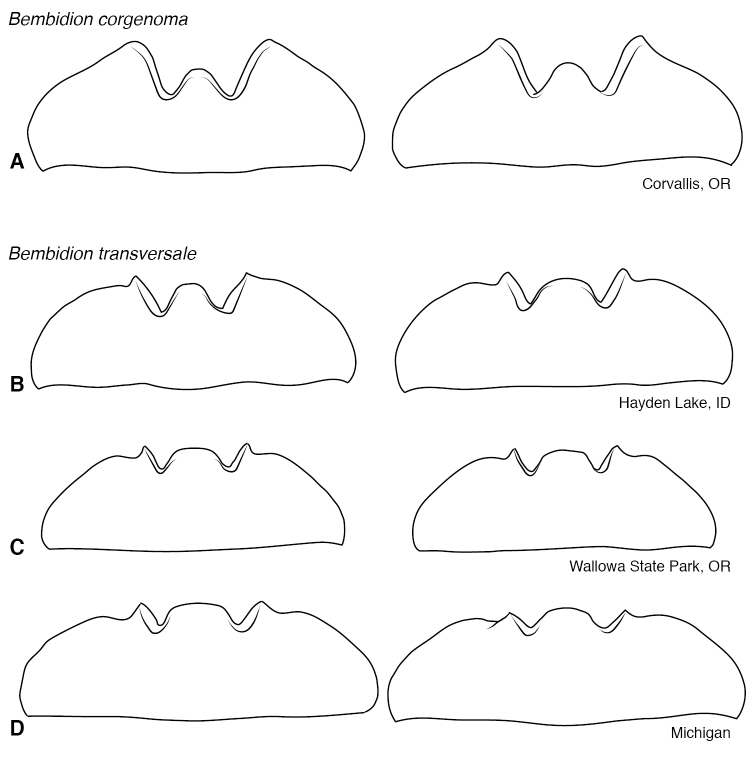
Mentum of *B. corgenoma* and *B. transversale***A** Two specimens of *B. corgenoma* from Corvallis, Oregon **B** Two specimens of *B. transversale* from Hayden Lake, Idaho **C** Two specimens of *B. transversale* from Wallowa State Park, Oregon **D** Two specimens of *B. transversale* from Point aux Pins, Michigan.

**Figure 14.**
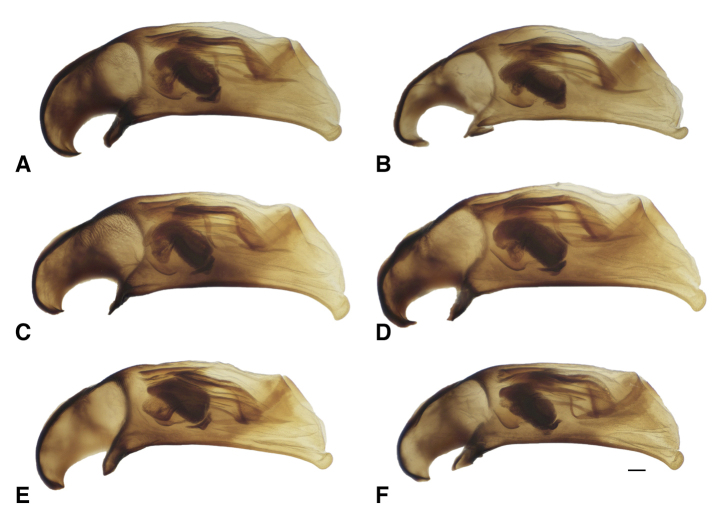
Male genitalia of *B. transversale* subgroup **A***B. transversale* (voucher DNA4219, USA: Oregon: Harney County, Banks of Silver Creek, 1379 m, 43.7278°N, 119.6256°W) **B***B. transversale* (voucher DNA2161, Canada: Alberta: Lethbridge, Oldman River, 800 m, 49.7043°N, 112.866°W) **C***B. erosum* (voucher DNA4033, USA: Oregon: Curry Co., Floras Creek at route 124 SE Langlois, 21 m, 42.9132°N, 124.4251°W) **D***B. erosum* (voucher DNA3562, USA: California: Del Norte Co., Wilson Creek, 3 m, 41.6051°N, 124.1005°W) **E***B. corgenoma* (voucher DNA2180, USA: California: Sonoma Co., Russian River, 3 mi NE Healdsburg) **F***B. corgenoma* (holotype, voucher DNA5673, USA: Oregon: Benton Co., Corvallis, Willamette River, 60 m, 44.5491°N, 123.2449°W). Scale bar: 0.1 mm.

**Figure 15.**
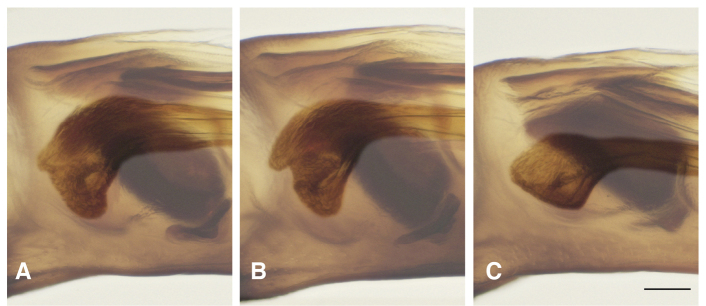
Basal sclerotized lobe of internal sac of male *Bembidion transversale* group members **A***B. transversale* (voucher DNA4219, USA: Oregon: Harney County, Banks of Silver Creek, 1379 m, 43.7278°N, 119.6256°W) **B***B. erosum* (voucher DNA4033, USA: Oregon: Curry Co., Floras Creek at route 124 SE Langlois, 21 m, 42.9132°N, 124.4251°W) **C***B. corgenoma* (voucher DNA2180, USA: California: Sonoma Co., Russian River, 3 mi NE Healdsburg). Scale bar: 0.1 mm.

**Figure 16.**
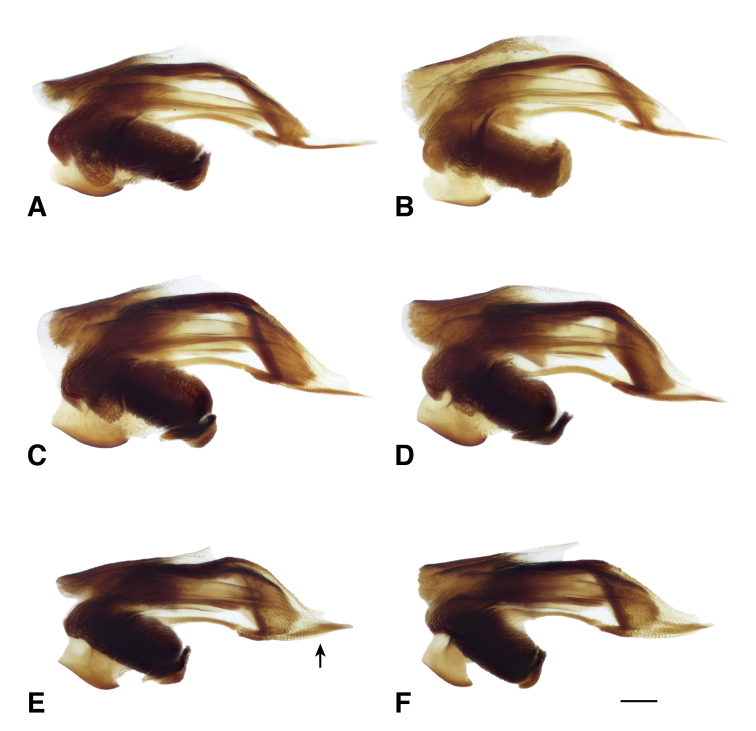
Central sclerite complex of *B. transversale* subgroup **A, B***B. transversale* (vouchers V101437 and V101436, USA: Michigan: Port aux Pins, Bois Blanc Isl.) **C, D***B. erosum* (vouchers V101440 and V101439, USA: California: Del Norte Co., Wilson Creek, 3 m, 41.6051°N, 124.1005°W) **E, F***B. corgenoma* (vouchers V101428 and V101430, USA: Oregon: Benton Co., Corvallis, Willamette River, 44.5475°N, 123.2428°W). Scale bar: 0.1 mm.

Adults of this species (Fig. 11A) are characterized by the reduced anterior lateral regions of the mentum (Fig. 13B–D), and the antenna with at least the second and third antennomeres apically infuscated (Fig. 12A). The prothorax is more cordate than in other species. The aedeagus has its ventral surface relatively straight, with the tip not abruptly curved downward (Fig. 14A, B); the basal sclerotized lobe is large (Fig. 15A), and the apex of the flagellar sheath has a long, thin dark line (Fig. 16A, B). This is the easternmost species, found from Newfoundland and Nova Scotia west through Ontario to southeastern British Columbia, central Oregon, northeastern Nevada, northern Utah, and Colorado (western portion of distribution shown in Fig. 19).

#### Bembidion erosum

Taxon classificationAnimaliaColeopteraCarabidae

(Motschulsky, 1850)

440E5220-8B61-5915-9AA4-7B17BBE8AAC8

Peryphus erosus Motschulsky, 1850:10. Lectotype female, in ZMUM, examined, designated by Bousquet and Larochelle (1993), labeled “type” [handwritten], “California” [handwritten on red paper], “Peryphus erosus Mots California” [handwritten on green paper], [a rectangle of blank red paper], “LECTOTYPE Peryphus erosus Motschulsky Des. by Y. Bousquet ’91” [partly handwritten on red paper]. Type locality California.
Bembidion marinicum Casey, 1918:57. Holotype female in USNM (type number 36919), examined. Type locality Marin County, California.

##### Diagnosis and geographic distribution.

Most adults of this species are the darkest members of this group (Fig. 11B), with the second and third antennomeres infuscated (Fig. 12B), at least apically, and with dark femora, although the more northern populations (e.g., from mainland British Columbia) have paler legs and paler ground color of the body. Prothorax moderately cordate, with a smooth basal region with few punctures. Tip of aedeagus abruptly curved downward (Fig. 14C, D); basal sclerotized lobe large (Fig. 15B); apex of flagellar sheath with long, thin dark line (Fig. 16C, D). This species is coastal, occurring from Haida Gwaii in British Columbia south along the coast to Big Sur and neighboring areas of California, with only one record from east of the Cascades (Fig. 20).

#### Bembidion corgenoma sp. nov.

Taxon classificationAnimaliaColeopteraCarabidae

03E7A18F-1F2E-5DA0-AA71-E569A3143062

http://zoobank.org/BF5E001D-F543-4149-8081-0BF7B99A8484

##### Holotype.

Male, in OSAC, labeled: “USA: Oregon: Benton Co., Corvallis, Willamette River, 60 m, 44.5491°N, 123.2449°W, 7.x.2019. DRM 19.210. D.R. Maddison”, “David R. Maddison DNA5673 DNA Voucher” [pale green paper], “HOLOTYPE Bembidion corgenoma David R. Maddison” [partly handwritten, on red paper], “Oregon State Arthropod Collection OSAC_0002000008 [matrix code]” [printed on both sides of white paper]. Genitalia mounted in Euparal in between coverslips pinned with specimen; extracted DNA stored separately. GenBank accession numbers for DNA sequences of the holotype are MW151449, MW151463, MW151491, MW151520, and MW151548.

##### Paratypes

**(193).** USA: Oregon: Benton Co., Corvallis, Willamette River, 44.5491°N, 123.2449°W, 60 m [type locality] (78: OSAC, CNC, CAS, UAIC, UASM, MCZ, EMEC, CSCA); USA: Oregon: Benton Co., Corvallis, Willamette River, 60 m, 44.5491°N, 123.2451°W (10: OSAC); USA: Oregon: Benton Co., Corvallis, Willamette River, 44.5475°N, 123.2428°W, 60 m (35: OSAC, USNM, NHMUK, MNHM, UBCZ); USA: Oregon: Benton Co., Corvallis, Willamette River, 60 m, 44.5478°N, 123.2430°W (2: OSAC); USA: Oregon: Benton Co., Corvallis, 62 m, 44.5491°N, 123.2449°W (6: OSAC); USA: Oregon: Linn Co., Willamette River, Truax Island, 44.5853°N, 123.1913°W, 60 m (12: OSAC); USA: Oregon: Lane Co., Goodman Creek, Willamette NF, 43.8441°N, 122.6736°W, 290 m (2: OSAC); USA: Oregon: Coos Co., Crooked Creek S of Bandon, 43.0814°N, 124.4335°W, 7 m, 24.iii.2014 (26: OSAC); USA: California: Tehama Co., Red Bluff, Sacramento River, 40.1759°N, 122.229°W, 73 m (22: OSAC, CAS, EMEC).

##### Type locality.

USA: Oregon: Benton Co., Corvallis, Willamette River, 44.5491°N, 123.2449°W.

##### Derivation of specific epithet.

The specific epithet is formed from the Latin word *cor*, meaning heart, and *genoma*, a modification (for easier pronunciation) of the coined word “genome”. *Corgenoma* refers to this species being the heart or current focus of genomic studies in small carabid beetles. *Cor*- also alludes to the type locality of Corvallis, Oregon, whose name is derived from Latin, and means “heart of the valley”. It is to be treated as a noun in apposition.

##### Diagnosis and description.

Length (5.8–7.4). Relatively light in color compared to *B. transversale* and *B. erosum*, with legs and basal three antennomeres pale, testaceous or rufo-testaceous. Head and prothorax piceous, with metallic reflections, on pronotum green or aeneous, on head bluish or green. Elytra paler, with shoulders and most of the anterior half testaceous with an orange tint, bordered posteriad by a dark band (with intervals 1–3 in this region dark rufous), followed by a pale testaceous band that either extends to the apex or that is bounded posteriad by dark lateral spots which in the darkest individuals merge in the middle. Mentum with anterior lateral region as typical for a *Bembidion*: triangular, large, and with anterior margin significantly anteriad of the central tooth (Fig. 13A); central tooth trapezoidal, rounded. Prothorax cordate, with more sinuate margins than *B. perspicuum*, with a relatively smooth basal region, with minute punctures; pronotum without or with very faint, oblique posterolateral carina as in other members of this group. Elytral striae 1–5 complete; stria 6 distinct and strong through much of its length; stria 7 shallower, less impressed than 6, but distinct. Microsculpture of elytra very transverse, with little tendency to form meshes. Two discal setae on each elytron, close to third stria. Tip of aedeagus not abruptly curved downward (Fig. 14E, F); basal sclerotized lobe small (Fig. 15C); apex of flagellar sheath with dark area triangular (Fig. 16E, F).

##### Additional characteristics.

Diploid chromosome number 24, with 11 pairs of autosomes and an XY/XX sex chromosome system (Pflug, et al. 2020). Genome size (1C), as measured by flow cytometry, 2118 Mb in males and 2193 Mb in females (Pflug, et al. 2020). Most specimens with singleton (non-conjugated) sperm (Gómez and Maddison 2020).

##### Available genomic and transcriptomic data.

Transcriptomic data for one specimen is available on NCBI’s Sequence Read Archive at accession SRR8801541, and genomic data of four specimens at accessions SRR8518612, SRR8518625, SRR8518626, and SRR8518631 (Pflug, et al. 2020).

##### Notes.

This species was called *Bembidion haplogonum* Chaudoir in Gustafson et al. (2019; 2020), and B. sp. nr. transversale in some other publications (Gómez and Maddison 2020; Kanda, et al. 2015; Pflug, et al. 2020).

##### Geographic distribution.

This species occurs from central British Columbia south through the Willamette Valley of Oregon, the Central Valley of California, with some records in Nevada, Idaho, eastern Washington, and Montana (Fig. 19), thus overlapping with the range of *B. transversale*.

##### Geographic variation.

The specimens on or close to the beaches of the Pacific Ocean appear on average slightly paler than more inland specimens.

##### Habitat.

This species occurs on gravel or cobble shores of the rivers and creeks (Fig. 4), more often where the bank is relatively flat and has small amount of clay and silt mixed in with sand and gravel under the rocks. They also can be common under cobbles on the shores of small creeks on the upper portions of beaches of the Pacific Ocean.

**Figure 17.**
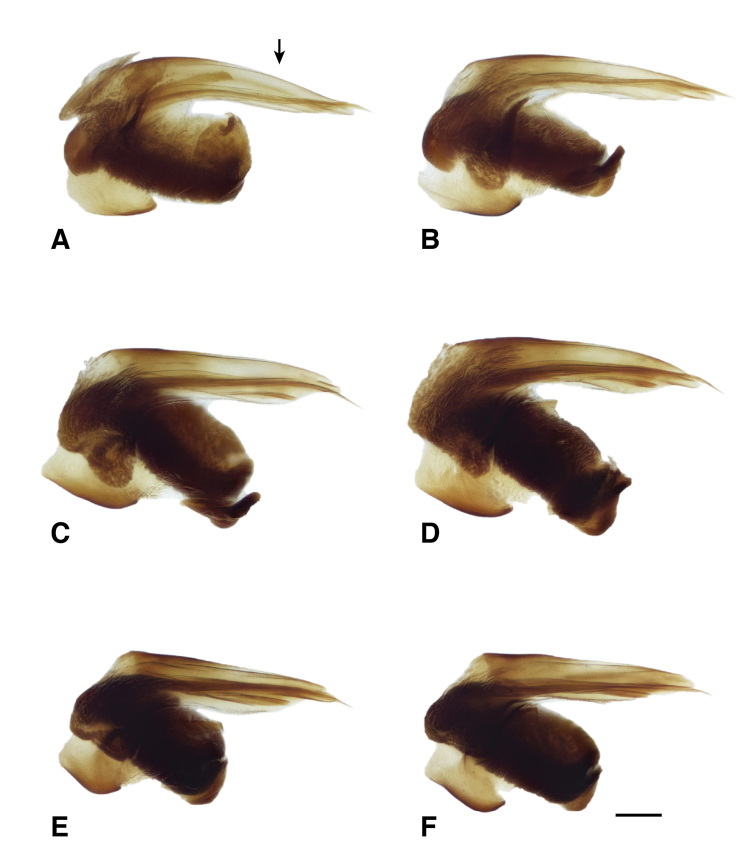
Flagella of *B. transversale* subgroup **A, B***B. transversale* (vouchers V101438 and V101435, USA: Michigan: Port aux Pins, Bois Blanc Isl.) **C, D***B. erosum* (vouchers V101442 and V101441, USA: California: Del Norte Co., Wilson Creek, 3 m, 41.6051°N, 124.1005°W) **E, F***B. corgenoma* (vouchers V101434 and V101431, USA: Oregon: Benton Co., Corvallis, Willamette River, 44.5475°N, 123.2428°W). Scale bar: 0.1 mm.

#### Bembidion perspicuum

Taxon classificationAnimaliaColeopteraCarabidae

(LeConte, 1848)

1E9155FF-00E0-54AE-95E0-60855C93F339

Ochthedromus perspicuus LeConte, 1848: 466. Holotype male, in MCZ (type number 5510), external structure and aedeagus examined. Type locality “Rocky Mountains”.
Ochthedromus mannerheimii LeConte, 1852:190. Lectotype female, designated by Maddison and Swanson (2010), in MCZ (type number 35571). Type locality San Diego, California. Although a male in the same unit tray as the lectotype is, by genitalic characters, a member of Bembidion corgenoma, I am uncertain about the lectotype. The almost complete absence of the seventh stria and the pronotal shape suggests Bembidion perspicuum, but the base of the pronotum is not as punctured as typical for that species. I tentatively place it as a synonym of Bembidion perspicuum. However, as Bembidion mannerheimii LeConte, 1852, is a junior homonym of Bembidion mannerheimii Sahlberg, 1827, this name is unavailable in any event.
Bembidium haplogonum Chaudoir, 1868: 241. Neotype male, in MNHN, here designated, labeled “USA: California: Lake Co., North Branch Cache Creek at hwy 20, 305 m 38.9881°N, 122.54°W, 5.viii.2010. DRM 10.090. K.W. Will & D.R. Maddison”, “David R. Maddison DNA5681 DNA Voucher” [pale green paper], “NEOTYPE Bembidium haplogonum Chaudoir designated D.R. Maddison” [partly handwritten, on white paper, bordered by red lines]. Genitalia mounted in Euparal in between coverslips pinned with specimen; extracted DNA stored separately. GenBank accession numbers for DNA sequences of the neotype are MW151478, MW151506, and MW151563. Details about the choice of neotype are provided below.
Bembidion acomanum Casey, 1918: 59. Lectotype female, designated by Lindroth (1975), in USNM (type number 36916), examined. Type locality Jemez Springs, New Mexico (Lindroth 1975).
Bembidion excursum Casey, 1918: 59. Holotype female, in USNM (type number 36915), examined. Type locality Tucson, Arizona.
Bembidion tuolumne Casey, 1924:30. Lectotype male, designated by Lindroth (1975), in USNM (type number 36917), external structure and aedeagus examined. In Maddison and Swanson (2010), this was treated as tentatively a synonym of B. transversale. Further examination of the lectotype, including of its genitalia, reveal that this is a specimen of B. perspicuum. Type locality Tuolumne, California.

##### Designation of a neotype for *Bembidium haplogonum* Chaudoir.

Lindroth (1963: 341) could not find the original type series for *Bembidium haplogonum* Chaudoir in the MNHN. Thierry Deuve and David Kavanaugh have both searched for it, and could not find it in the Chaudoir collection, although other specimens were found that had been collected in California by Pierre Joseph Michel Lorquin. As the original type series is presumed lost, I here designate a neotype.

Chaudoir’s (1868) description of *Bembidium haplogonum* is detailed enough to make determination of the species he was describing clear. In his description, the large size (8 mm) and absence of a carina near the hind angle of the prothorax could only apply (within California, the type locality) to a member of the *B. transversale* group. The color pattern of the elytra (“*sur les élytres, une petite tache au milieu du bord antérieur de chacune, et une bande transversale un peu arquée aux trois quarts, d’un jaune testacé pâle, peu distinctes, surtout la tache basale*”, which translates to “on the elytra, a small spot on the front edge of each, and a slightly curved transverse band at three-quarters, of a pale testaceous yellow, indistinct, especially the basal spot”) can only apply to some specimens of *B. erosum*, *B. lugubre*, or the northern, dark form of *B. perspicuum* (Fig. 11E), as the other species in California have the entire basal third to half of the elytra pale. The description of the appendages as having the first 3.5 antennomeres, palps, and legs all pale yellow eliminates *B. erosum*, as the palps, femur, and antennomeres 2–11 are dark in California specimens of that species. I have seen no specimens of *B. lugubre* with an isolated small spot on the front edge of each elytron; the only specimens that have a pale area in the basal half of the elytra have the entire sides and basal regions of the elytra a pale rufous, with a darker disc; this paler form of *B. lugubre* occurs north of Los Angeles in California. The metallic coloration of the pronotum (“*Le dessus d’un vert brillant un peu cuivreux*”) is characteristic of *B. perspicuum*, but not *B. lugubre*; the latter has no metallic sheen in any specimens I have seen from California, and in only very few individuals elsewhere. The flatter prothorax with less rounded sides and a wider lateral gutter is also distinctive of *B. perspicuum* relative to all three other species from California (*B. erosum*, *B. lugubre*, and *B. corgenoma*), as is the distinctly punctured pronotal base (“*distinctement ponctué tout le long de la base*”). The large size (8 mm) is more characteristic of *B. perspicuum*; I have seen no specimens of *B. corgenoma* longer than 7.5 mm, but have seen specimens of *B. perspicuum* that are 7.9 mm in length, and specimens of *B. perspicuum* are, in general, larger than those of *B. corgenoma*. Finally, the virtual absence of the seventh elytral stria is characteristic of *B. perspicuum* relative to *B. corgenoma*. As Lorquin travelled extensively in areas where the dark form of *B. perspicuum* occurs (Grinnell 1904), it is certainly reasonable that a specimen of that form could have been seen by Chaudoir.

My early interpretations of Chaudoir’s descriptions were in error, and led me to believe that Chaudoir’s specimen was a member of what I here call *Bembidion corgenoma*; that mistake led me to call the species studied in Gustafson et al. (2019)*Bembidion haplogonum*. As a correct reading of the original description shows that *Bembidium haplogonum* refers to the dark form of *B. perspicuum*, I have designated a specimen from northern California with a color pattern matching Chaudoir’s description as the neotype (shown in Fig. 11E).

##### Diagnosis and geographic distribution.

Adults of this species are large, and have a pronotum that is flatter than in other members of the group, with less rounded sides, and with the basal region more evidently punctate (Maddison and Swanson 2010: fig. 4). At least antennomeres 4–11 infuscated. Specimens from most areas are relatively pale (Fig. 11D), with the front half of the elytra pale, but specimens from northern California and Oregon are much darker (Fig. 11E), with only elytral apices being pale. Aedeagal characterс are described in Maddison and Swanson (2010). This species is known from Texas, Kansas, Colorado, New Mexico, Arizona, Utah, Nevada, California, and Oregon.

#### Bembidion sarpedon

Taxon classificationAnimaliaColeopteraCarabidae

Casey, 1918

21CB526F-633B-5C12-9B17-A59B878AABAD

Bembidion sarpedon Casey, 1918: 58. Lectotype male, designated by Lindroth (1975), in USNM (type number 36914); external structure and aedeagus examined. Type locality Cañon City, Colorado.
Bembidion animatum Casey, 1918: 62. Lectotype female, designated by Lindroth (1975), in USNM (type number 36918), examined. Type locality Jemez Springs, New Mexico (Lindroth 1975).

##### Diagnosis and geographic distribution.

Adults of this species (Fig. 11F) are the palest members of this group, with legs entirely testaceous or rufo-testaceous, with antennae gradually becoming slightly darker toward the apex, and pronotum in most specimens dark rufous as opposed to the piceous or black of other species. The dorsal surface is shinier than in other species, especially the pronotum, because of the nearly effaced microsculpture. The prothorax is moderately cordate; the elytral intervals are flatter than in related species, with small punctures in the striae. The ventral margin of the aedeagus has a slight downward bulge, and the internal sac sclerite complex of male genitalia is narrow in lateral view, with a long and thin flagellar complex. Known from New Mexico and Colorado west to Arizona and Utah, and north to Mammoth Hot Springs, Wyoming (OSAC).

## Concluding remarks

The pathways that led to the recognition of the two species described here were very different. When I encountered *Bembidion mimbres* for the first time, as pinned specimens at the University of Alberta’s Strickland Museum in 1981–1982, I immediately recognized them as an undescribed species. They shared the large size, setose elytra, shiny surface, and striking color of the distinctive *Bembidion levigatum*, but did not share *B. levigatum*’s unusual prothorax shape and width.

In contrast, it took at least 12 years of study for me to become confident that *B. corgenoma* was a new species, and that the *B. transversale* subgroup consisted of at least five species (*B. sarpedon*, *B. perspicuum*, *B. transversale*, *B. erosum*, and *B. corgenoma*). The distinctiveness of *B. sarpedon* and *B. perspicuum* was recognized many years ago. The specimens that remained (*B. transversale* s. l.), however, were so complex in their variation patterns, so lacking in a differentiating signal in DNA sequences, and with such similar genitalia, that at times I thought there was just one species in *Bembidion transversale* s. l., and at other times more.

**Figure 18.**
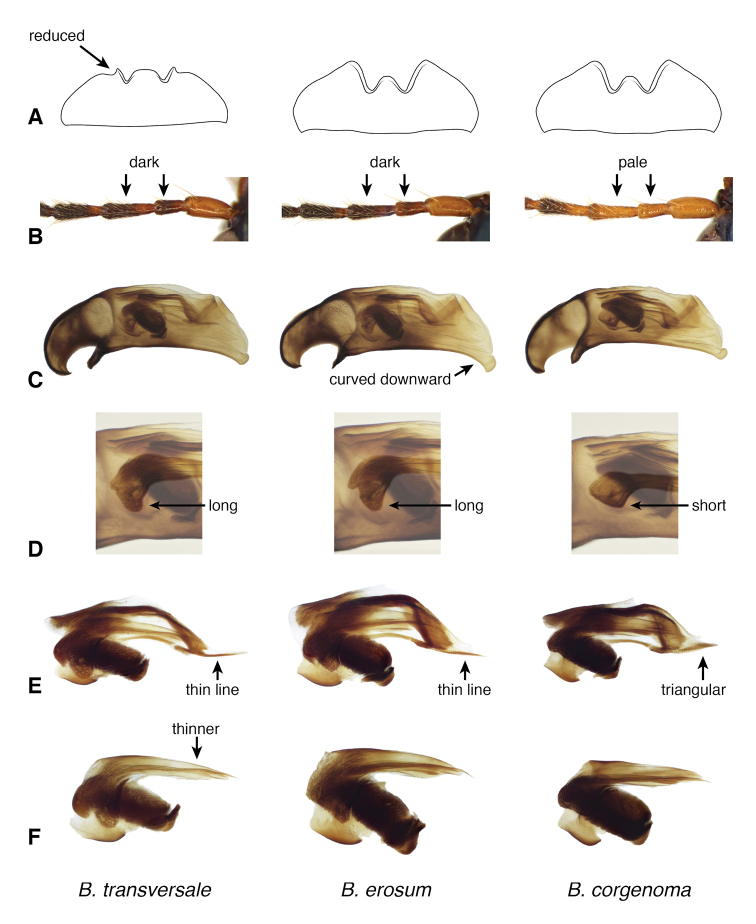
Summary of morphological differences between three species in the *B. transversale* subgroup in **A** mentum **B** antennal color **C** curvature of ventral margin of the aedeagus **D** basal sclerotizes lobe size **E** apex of flagellar sheath, **F** flagellum.

I had become so accustomed to the clarity provided by DNA sequences in my other taxonomic projects on bembidiines that I had become somewhat skeptical of the value of traditional taxonomic methods utilizing only patterns of morphological variation. Two events changed my mind, as they caused the patterns to become evident at last. The first event was Kip Will’s collecting of both dark and light specimens from the shores of Wilson Creek in north coastal California. They were so obviously different in color that I expected them to have clearly different genitalia, and different DNA sequences. My cursory inspection revealed only the slightest difference in the overall shape of the aedeagus (I had not yet noticed the differences in the structures of the internal sac), not notable enough to be significant in itself. In addition, all six sequenced dark specimens from that gravel bar differed from all five sequenced light specimens in one base in Topoisomerase, but they did not differ in 28S, COI, and CAD. The correlation between color, aedeagal shape, and that single base in Topoisomerase convinced me that there were likely two species at that site in northern California, although if so they would be much more similar than are most other pairs of closely related, sympatric *Bembidion* species. Examination of Motschulsky’s specimens eventually revealed that the dark species had a name, *Bembidion erosum*, but the pale species at Wilson Creek and elsewhere continued to trouble me: other than the normal mentum, I saw no consistent differences from the more eastern *Bembidion transversale*. The distinctiveness of the pale western form (here called *B. corgenoma*) did not become evident until the basic morphological work was done: thorough examination of the genitalia of 63 *B. corgenoma* males and 33 *B. transversale* males, focused on the area of geographic overlap, revealed the consistent differences shown in Fig. 18, especially the shape of the basal sclerotized lobe (Fig. 18D) and the tip of the flagellar sheath (Fig. 18E). This confirmed that sequences of four genes will not necessarily reveal the presence or absence of gene flow, and that even in *Bembidion*, a group in which DNA sequences often work very well for species delimitation, species boundaries are sometimes more quickly uncovered by traditional morphological methods.

The lack of observed differentiation in DNA sequences between *Bembidion transversale*, *B. erosum*, and *B. corgenoma* suggests that these are young, recently differentiated species. The contrast is striking between this trio and other bembidiines; in most bembidiines, sequences in at least one of the handful of standard genes provides a clear signal of lack of gene flow between species (e.g., Maddison 2008; Maddison and Cooper 2014; Maddison and Sproul 2020; Sproul and Maddison 2017). Why is the signal of species boundaries so clear in most bembidiine groups, but not *B. transversale* s. l.? Given genomic resources now available for this group, one fruitful and available avenue of future research would be comparison of coalescent patterns of thousands of regions of the genome within both this trio of *Bembidion transversale* group species and other groups of bembidiines with similar levels of morphological divergence.

**Figure 19.**
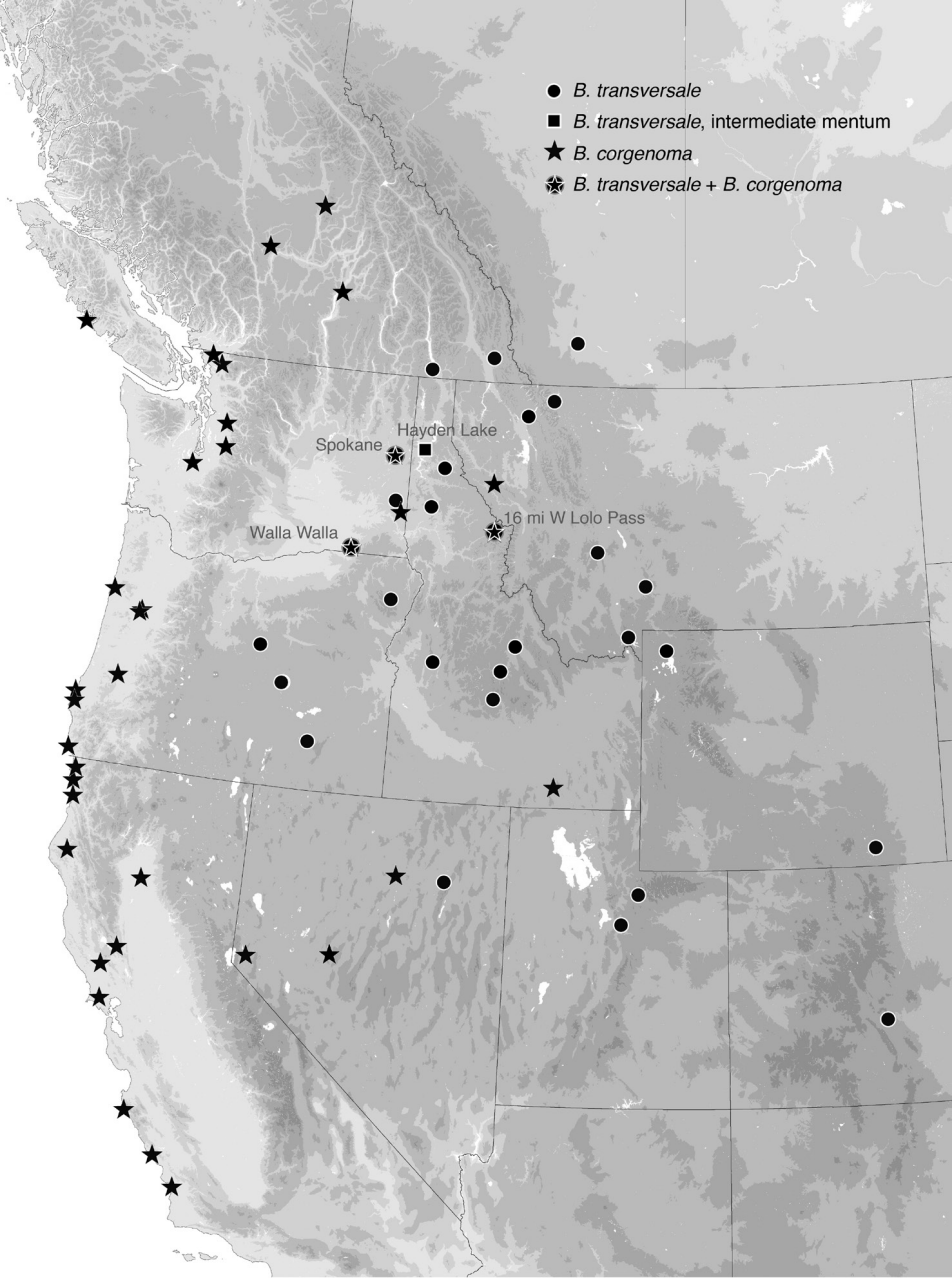
Geographic distribution of *B. transversale* and *B. corgenoma* (eastern portion of distribution of *B. transversale* not shown).

**Figure 20.**
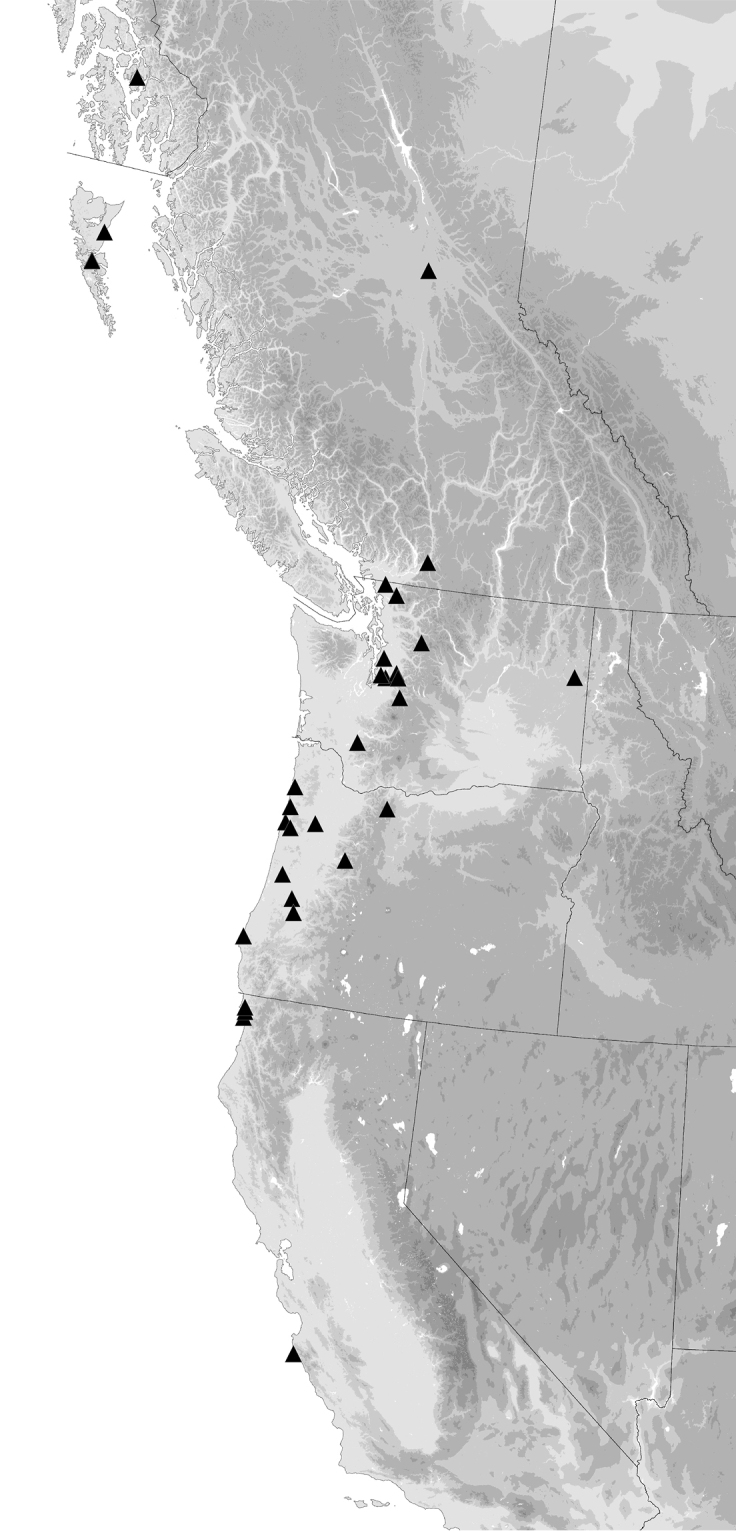
Geographic distribution of *B. erosum*.

## Supplementary Material

XML Treatment for Bembidion mimbres

XML Treatment for Bembidion levigatum

XML Treatment for Bembidion transversale

XML Treatment for Bembidion erosum

XML Treatment for Bembidion corgenoma

XML Treatment for Bembidion perspicuum

XML Treatment for Bembidion sarpedon

## Supplementary materials

Supplementary material 1 Mesquite NEXUS file containing the DNA sequence data and resulting phylogenetic trees from maximum likelihood analyses for the subgenus Hydrium Data type NEXUS file (DNA sequences, phylogenetic trees) https://binary.pensoft.net/file/492385This dataset is made available under the Open Database License (http://opendatacommons.org/licenses/odbl/1.0/). The Open Database License (ODbL) is a license agreement intended to allow users to freely share, modify, and use this Dataset while maintaining this same freedom for others, provided that the original source and author(s) are credited.David R. Maddison

Supplementary material 2 Mesquite NEXUS file containing the DNA sequence data and resulting phylogenetic trees from maximum likelihood analyses for the *Bembidion transversale* group Data type NEXUS file (DNA sequences, phylogenetic trees) https://binary.pensoft.net/file/492386This dataset is made available under the Open Database License (http://opendatacommons.org/licenses/odbl/1.0/). The Open Database License (ODbL) is a license agreement intended to allow users to freely share, modify, and use this Dataset while maintaining this same freedom for others, provided that the original source and author(s) are credited.David R. Maddison
